# Iatrogenic Pseudoaneurysm After Arthroscopic Knee Procedures: A Systematic Review of Presenting Features, Injured Vessels, Management, and Outcomes

**DOI:** 10.7759/cureus.116501

**Published:** 2026-09-18

**Authors:** Ethan J Sack, Max Deneroff, Zubair Mohammed, Austin Khau, Chadi Ellaham, Jared W Nichols

**Affiliations:** 1 Medicine, Kansas City University of Medicine and Biosciences, Joplin, USA; 2 College of Osteopathic Medicine, Kansas City University, Joplin, USA; 3 Osteopathic Manipulative Medicine/Family Medicine, Kansas City University, Joplin, USA

**Keywords:** acl reconstruction, ct angiography, doppler ultrasound, false aneurysm, genicular artery, hemarthrosis, knee arthroscopy, meniscectomy, popliteal artery, pseudoaneurysm

## Abstract

Pseudoaneurysm after arthroscopic knee procedures is uncommon but clinically important because it can initially resemble routine postoperative swelling, hematoma, recurrent hemarthrosis, deep vein thrombosis, infection, or compartment syndrome. PubMed, Embase, and Scopus were searched through July 9, 2026. The original database pathway yielded 312 records and 54 included reports representing 60 patient cases. During peer-review citation audit, three additional potentially eligible reports were identified; two full texts were retrieved and included, while one report could not be retrieved and was not used for patient-level inference. The final synthesis therefore included 56 reports and 62 patient cases. Using a mutually exclusive primary-procedure classification, meniscal procedures accounted for 25/62 cases (40.3%) and anterior cruciate ligament (ACL)-related procedures for 19/62 (30.6%). The popliteal artery was the most frequent injured-vessel group (26/62, 41.9%), followed by medial/inferior genicular branches (9/62, 14.5%). Among 49 cases with an explicitly quantifiable interval to vascular diagnosis or recognition, the median was 14 days (range: 1-365 days); 13 cases had missing or non-quantifiable timing. Primary treatment was open vascular surgery in 30 cases, endovascular embolization/coiling in 14, ultrasound-guided thrombin injection in six, covered-stent or other endovascular repair in five, conservative observation in two, an unspecified vascular approach in four, and no definitive pseudoaneurysm-directed treatment was reported in one. Fasciotomy and other adjunctive procedures were coded separately. Final outcome categories were favorable recovery/resolution without persistent morbidity in 42 cases, persistent neurologic/functional morbidity or incomplete recovery in four, limited or short-term follow-up in 10, and no outcome reported in six. These frequencies describe published cases only and cannot estimate incidence, risk, or comparative treatment effectiveness. Recurrent or progressive postoperative symptoms, a pulsatile finding, neurologic change, or symptoms out of proportion to the expected course should prompt consideration of vascular imaging.

## Introduction and background

Knee arthroscopy is widely used for meniscal, ligamentous, synovial, and diagnostic pathology. Early postoperative pain, bruising, stiffness, and swelling are common and can obscure an arterial injury. A pseudoaneurysm forms when an arterial wall defect remains in communication with the parent vessel but is contained by surrounding tissue; after arthroscopy, it may present later as recurrent hemarthrosis, hematoma, focal swelling, or symptoms that mimic venous thrombosis or infection [1-4].

The anatomy around the knee creates risk to both the popliteal artery and smaller periarticular branches. Posterior meniscal work, posterior capsular instrumentation, portal placement, synovectomy, cyst procedures, and adjacent soft-tissue dissection have all been linked to injuries involving the popliteal, genicular, descending genicular, sural, or nearby muscular branches [5-11].

Ligament reconstruction adds procedure-specific mechanisms such as hamstring harvest, tibial or femoral tunnel drilling, fixation hardware, outside-in trajectories, revision fixation, and combined cruciate reconstruction. The reported anterior cruciate ligament (ACL)-related cases therefore span both main-vessel and branch-vessel injury patterns rather than a single mechanism [12-20].

Clinical presentation is equally variable. Some patients have a classic pulsatile mass, bruit, or thrill, whereas others first develop persistent pain, recurrent hemarthrosis, calf swelling, neurologic deficit, suspected deep vein thrombosis, or a compartment-syndrome-like picture [21-24]. Distal pulses may remain present in branch-vessel injury or with preserved collateral flow, so a normal pulse examination does not exclude pseudoaneurysm.

Imaging is central when a vascular complication is suspected. Duplex or Doppler ultrasound can identify arterial flow in accessible lesions, CT angiography can define deeper anatomy and competing diagnoses, and catheter angiography can confirm the injured vessel while permitting embolization in selected cases [25-29]. These modalities play different clinical roles and should not be interpreted as interchangeable first-line tests in every patient.

Management in the published cases includes open resection, ligation, primary repair, patch angioplasty, interposition grafting, and bypass for selected injuries [30-37]. Branch-vessel pseudoaneurysms have also been managed with coil or liquid embolization and ultrasound-guided thrombin injection, while selected main-vessel injuries have been treated endovascularly [38-42]. The case literature is too heterogeneous to establish a single best treatment or a validated treatment hierarchy.

The knowledge gap is therefore not whether pseudoaneurysm can occur after knee arthroscopy, but how the scattered case literature can be organized into clinically useful patterns without implying population-level incidence or comparative effectiveness. This review summarizes the procedures involved, injured vessels, warning signs, diagnostic pathways, treatments, and outcomes while explicitly separating mutually exclusive from overlapping descriptive categories.

## Review

Methods

Study Design and Protocol

We conducted a descriptive systematic review of published case reports and small case series describing pseudoaneurysm after arthroscopic knee procedures, reported in accordance with PRISMA 2020 guidance [43]. A formal review protocol was not prospectively registered or publicly deposited. The clinical review question, eligibility criteria, patient-level extraction approach, and descriptive synthesis were established before the final synthesis. Revisions made after peer review consisted of a targeted citation audit, inclusion of two newly retrieved eligible reports, design-matched appraisal, and recoding of procedure, vessel, treatment, imaging-role, and outcome variables for clarity; the clinical eligibility question was not changed.

Search Strategy and Data Sources

Initial searches were performed in May 2026, and the final database searches of PubMed, Embase, and Scopus were completed on July 9, 2026. No publication-date restriction was applied. The exact platform-specific strategies are provided in Table 1. The syntax was not made literally identical across platforms because PubMed and Embase use controlled vocabulary whereas Scopus does not; however, the core concepts of false aneurysm/pseudoaneurysm, knee arthroscopy, and relevant knee vascular anatomy were used across all three sources. The displayed strategies are the actual final searches and were not retroactively rewritten after peer review.

**Table 1:** Full database search strategies

Database	Exact final strategy	Records identified
PubMed	(("Aneurysm, False"[Mesh] OR pseudoaneurysm*[tiab] OR "false aneurysm"[tiab] OR "false aneurysms"[tiab]) AND ("Arthroscopy"[Mesh] OR arthroscop*[tiab] OR "knee arthroscopy"[tiab]) AND ("Knee Joint"[Mesh] OR knee[tiab] OR popliteal[tiab] OR genicular[tiab] OR sural[tiab]))	82
Embase	('false aneurysm'/exp OR pseudoaneurysm*:ti,ab,kw OR 'false aneurysm':ti,ab,kw) AND ('knee arthroscopy'/exp OR 'arthroscopy'/exp OR arthroscop*:ti,ab,kw) AND ('knee'/exp OR knee:ti,ab,kw OR popliteal:ti,ab,kw OR genicular:ti,ab,kw OR sural:ti,ab,kw)	106
Scopus	TITLE-ABS-KEY(pseudoaneurysm* OR "false aneurysm" OR "false aneurysms") AND TITLE-ABS-KEY(arthroscop* OR meniscectom* OR "meniscal repair" OR "meniscus repair" OR "ACL reconstruction" OR "anterior cruciate ligament reconstruction" OR "PCL reconstruction" OR "posterior cruciate ligament reconstruction" OR synovectom* OR debridement) AND TITLE-ABS-KEY(knee OR popliteal OR genicular OR sural OR "descending genicular" OR "deep femoral")	124
Total	PubMed + Embase + Scopus	312

Language restrictions were not encoded in the electronic searches. Among full-text reports assessed for eligibility, zero were excluded solely for language, and no otherwise-eligible full text required translation. Reference lists of included articles and prior reviews were also examined for older reports. The contemporaneous review log did not preserve a separate numeric count for initial reference-list searching, so no retrospective hand-search count was imputed. During peer review, however, a targeted citation audit identified three specific potentially eligible reports; two were retrieved and included and one could not be retrieved. The database sources, final search dates, and numbers of records identified are summarized in Table 2.

**Table 2:** Database sources and raw record counts

Database	Final search date	Records identified
PubMed	July 9, 2026	82
Embase	July 9, 2026	106
Scopus	July 9, 2026	124
Total		312

The exact final search strategies used for PubMed, Embase, and Scopus are presented in Table 1.

Deduplication and Study Selection

Records were consolidated in an Excel-compatible screening spreadsheet. No dedicated automated deduplication program was used. Exact DOI matches and exact-title matches were removed first; near-duplicates were then adjudicated manually using title, author, publication year, journal, and article content when necessary. The high duplicate proportion reflects deliberate overlap among broad PubMed, Embase, and Scopus searches. Six reports removed at the full-text stage represented duplicate patient reports or duplicate full-text copies that could only be recognized reliably after article-level comparison; they were therefore distinct from the record-level duplicates removed before title/abstract screening. Two reviewers screened titles/abstracts and independently assessed potentially eligible full texts, resolving disagreements by discussion and consensus.

Eligibility Criteria

Eligible publications were human case reports or case series with extractable patient-level data describing a pseudoaneurysm that followed an arthroscopic knee procedure. The operational category of “other therapeutic knee arthroscopy” required an intra-articular or portal-based arthroscopic intervention temporally preceding the vascular lesion; it included arthroscopic synovectomy, irrigation/debridement, cyst procedures, and similar therapeutic arthroscopy. Procedures that were primarily open, arthroplasty-based, trauma-only, non-knee, or non-arthroscopic were excluded. Associated arteriovenous fistula was allowed when a pseudoaneurysm was also present. The operational inclusion and exclusion criteria are summarized in Table 3.

**Table 3:** Operational inclusion and exclusion criteria ACL: Anterior cruciate ligament; PCL: posterior cruciate ligament; AVF: atriovenous fistula

Inclusion criteria	Exclusion criteria
ACL reconstruction/repair or ACL-related arthroscopy	Total or revision knee arthroplasty
Arthroscopic meniscectomy or meniscal repair/root repair	Open or other non-arthroscopic knee surgery
PCL or multiligament arthroscopic reconstruction	Trauma-only pseudoaneurysm unrelated to arthroscopy
Diagnostic knee arthroscopy	Non-knee arthroscopy
Arthroscopic synovectomy, irrigation/debridement, cyst procedure, or other portal-based therapeutic arthroscopy	True aneurysm without postoperative arterial injury
Pseudoaneurysm with associated AVF when related to knee arthroscopy	Isolated AVF without pseudoaneurysm
Case reports/series with patient-level data	Review-only publications, duplicate patient reports, or reports without patient-level data

Data Extraction, Coding, and Synthesis

Two reviewers independently extracted patient-level data using a standardized spreadsheet, with one row per patient whenever cases could be separated. Extracted variables included demographics, comorbidity/coagulopathy, procedure, surgical details, injured vessel, presentation, timing, imaging, treatment, outcome, recurrence, neurologic or ischemic sequelae, and other complications. Unreported fields were coded as “Not reported” rather than inferred.

Procedure was recoded into one mutually exclusive primary category per case: meniscal; isolated ACL-related; combined ACL and meniscal; PCL/combined cruciate; synovectomy/debridement/irrigation/cyst procedure; or other diagnostic/therapeutic arthroscopy. Raw vessel wording was preserved in the extraction dataset, while a standardized vessel group was assigned for synthesis. When a source used unusual terminology, such as “superior middle genicular artery” reported by Becher et al. [4], the original wording was retained and grouped under a broader superior medial/middle genicular category rather than silently rewritten.

One mutually exclusive primary vascular-treatment category was assigned per case; adjunctive procedures such as fasciotomy, hematoma evacuation, and nerve reconstruction were coded separately. Outcomes were recoded into four mutually exclusive final-status categories: favorable recovery/resolution without persistent morbidity documented; persistent neurologic/functional morbidity or incomplete recovery; limited/short-term follow-up in which definitive recovery was not established; and outcome not reported. Imaging was retained at the patient level with role notes. Diagnostic vascular ultrasound was distinguished from ultrasound used only for aspiration, procedural guidance, or follow-up, and catheter angiography was distinguished conceptually from noninvasive initial imaging because it was frequently confirmatory, treatment-planning, or treatment-associated.

For time-to-diagnosis analysis, an interval in days was coded only when the source provided an explicit postoperative interval to vascular diagnosis/recognition. When the exact diagnosis date was not separately stated, the first explicit recognition of the pseudoaneurysm was used. Vague intervals such as “several weeks” or “several months” were excluded from the numerical calculation rather than converted. The coding definitions and grouping rules used for the descriptive synthesis are detailed in Table 4.

**Table 4:** Coding dictionary and prespecified grouping rules ACL: Anterior cruciate ligament; PCL: posterior cruciate ligament

Primary procedure	One mutually exclusive group per patient: meniscal; ACL-related; combined ACL+meniscal; PCL/combined cruciate; synovectomy/debridement/irrigation/cyst; other diagnostic/therapeutic arthroscopy.
Vessel	Raw source wording preserved. Standardized group assigned by named injured vessel/branch. Atypical source wording such as “superior middle genicular artery” is retained verbatim in raw data and grouped under superior medial/middle terminology.
Treatment	One mutually exclusive primary vascular treatment per case. Fasciotomy, hematoma evacuation, nerve procedures, and compression attempts are adjunctive variables and do not inflate the primary-treatment count.
Outcome	One mutually exclusive final-status category: favorable recovery/resolution without persistent morbidity; persistent neurologic/functional morbidity/incomplete recovery; limited/short-term follow-up; not reported.
Time to diagnosis	Numerically coded only for explicit intervals. First definite pseudoaneurysm recognition used when diagnosis date not separately stated. Vague intervals left missing.
Diagnostic vascular ultrasound	Counted only when ultrasound/Doppler/duplex evaluated the vascular lesion. Aspiration-only, procedural-guidance-only, or follow-up-only ultrasound is separated.
Angiography role	Patient-level role notes distinguish primary/definitive angiography from confirmatory, planning, or treatment-associated angiography when sequence is reported.
Missing information	Coded as “Not reported.” Missing source information is not inferred from indirect or incomplete evidence

Methodological Appraisal

Methodological appraisal was matched to publication design using JBI critical appraisal tools [44]. Single-patient reports were considered with the JBI Case Reports checklist, while the six multi-patient publications were considered with the JBI Case Series checklist. Responses were retained as Yes, No, Unclear, or Not applicable. No numerical quality score was calculated, appraisal did not determine eligibility, and “Unclear” or “Not applicable” responses were not collapsed into “No.” The revised study-selection flow diagram was prepared in a PRISMA 2020-compatible format [45]. The methodological appraisal approach and the JBI checklist used for each publication design are summarized in Table 5.

**Table 5:** Methodological appraisal approach JBI: Joanna Briggs Institute

Publication design	JBI tool	Number of reports	Reporting approach
Single-patient case report	JBI Case Reports checklist	50	Y/N/U/NA; no score
Multi-patient report/case series	JBI Case Series checklist	6	Y/N/U/NA; no score

Statistical Approach

Because the evidence consisted of case reports and small case series without comparator groups, standardized follow-up, or uniform outcome definitions, meta-analysis and comparative effectiveness testing were not appropriate. Findings are reported with counts, percentages, medians, and ranges. Wilson confidence intervals were removed because the published cases are a selected, publication-biased sample and interval estimates could imply population-level precision that the design cannot support. No P-values, subgroup hypothesis tests, or historical-period comparisons were performed.

Results

Study Selection

The three database searches identified 312 raw records. After 237 record-level duplicates were removed, 75 records underwent title/abstract screening; 14 were excluded. Sixty-one full-text reports were assessed, of which seven were excluded: six duplicate patient reports or duplicate full-text copies and one trauma-only/non-arthroscopic report. The original database pathway therefore contributed 54 reports representing 60 patient cases. The original corpus included both older and contemporary case-based reports across meniscal, ligament, synovial, and other arthroscopic settings [46-57]. The Preferred Reporting Items for Systematic Reviews and Meta-Analyses (PRISMA) flowchart is shown in Figure 1.

**Figure 1:**
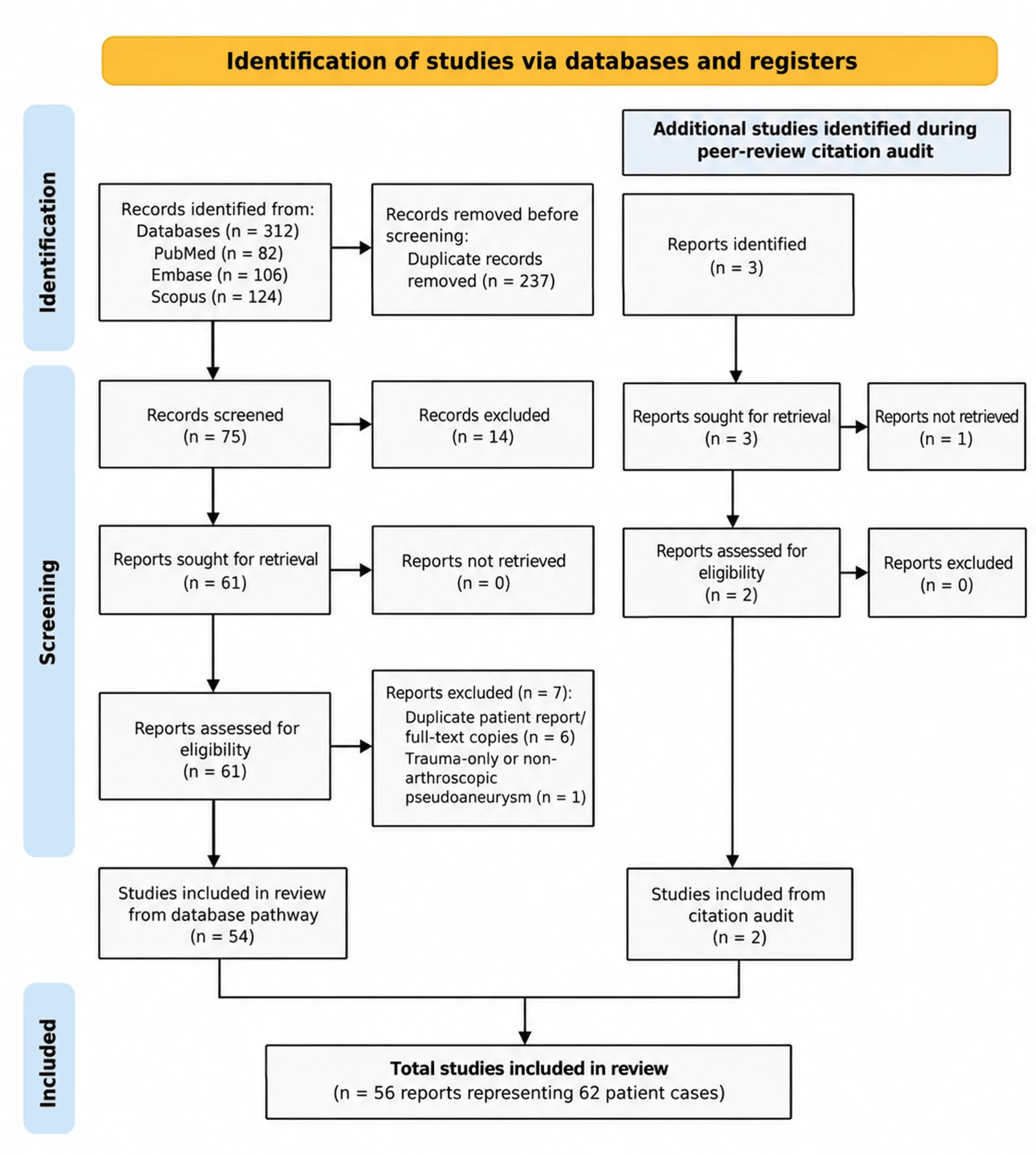
PRISMA 2020 flow diagram PRISMA: Preferred Reporting Items for Systematic Reviews and Meta-Analyses

During peer review, three additional potentially eligible publications were specifically identified. Full texts for Armato and Czarnecki and for Milankov et al. were retrieved and met the prespecified eligibility criteria, adding one patient each [58,59]. The third peer-review-identified publication (Pereira Junior et al., 2010) could not be retrieved; because patient-level eligibility and data could not be verified, it was not included and no patient data were inferred from secondary descriptions. The final synthesis therefore contains 56 reports and 62 patients. The retrieval status and inclusion decisions for the three reports identified during the peer-review citation audit are summarized in Table 6.

**Table 6:** Peer-review citation audit

Report identified during peer review	Retrieval	Disposition
Armato & Czarnecki (1990), Geniculate artery pseudoaneurysm: a rare complication of arthroscopic surgery	Full text retrieved	Included; one patient added [58]
Milankov et al. (2006), Pseudoaneurysm of the medial inferior genicular artery following ACL reconstruction with hamstring tendon autograft	Full text retrieved	Included; one patient added [59]
Pereira Junior et al. (2010), described by reviewer as two pseudoaneurysms after arthroscopic knee procedures	Full text not retrieved	Not included; patient-level eligibility/data not inferred

Study Characteristics and Demographics

Age was reported in 60/62 patients. Exact numeric age was available in 59/62 and was used for the age summary; the median was 31 years (range, 8-84 years). One additional report described age only as being in the patient's 20s and was not assigned a numeric value. Sex was reported in 60/62 patients: 46 were male and 14 were female. The review spans several decades of case literature, but no era-stratified statistical comparison was performed and no temporal treatment trend is inferred. Patient-level data for the 62 included cases are presented in Table 7.

**Table 7:** Complete patient-level extraction

Study_ID	Case_ID	Source_PDF	First_author	Year	Country	Journal	Title	Article_type	Review_paper_note	Age	Sex	Comorbidities_anticoagulation_coagulopathy	Procedure_category	Procedure_details	Surgical_details_graft_fixation_tunnel_portals_tourniquet	Injured_vessel	Presentation	Time_to_diagnosis	Imaging	Treatment	Outcome	Complications	Extraction_status	Confidence	Notes	Primary_procedure_group	Standardized_vessel_group	Primary_treatment_group	Adjunctive_treatment	Outcome_category	Time_to_diagnosis_days	Imaging_role_notes
01	01	1. Pseudoaneurysm of the Superficial Femoral Artery after Knee Arthroscopy.pdf	Carroll	2020	Ireland	Cureus	Pseudoaneurysm of the Superficial Femoral Artery after Knee Arthroscopy	Case report	No	84	M	Atrial fibrillation on apixaban; Hodgkin lymphoma; TIA; hypertension	Meniscectomy/debridement	Knee arthroscopy for severe OA; debridement of degenerative meniscus	Tourniquet mentioned; indirect/tourniquet-related SFA injury suspected	Superficial femoral artery	Lower-limb swelling and pain; DVT-like presentation	3 weeks postop	Ultrasound venogram; CT lower-limb angiogram	Covered stent	Pain resolved/dissipated; recovered after vascular treatment		Full-text audit completed against available full text; unresolved fields marked Not reported	High		Meniscal procedure	Superficial femoral/vastus branch	Covered stent/stent-graft	None separately reported	Favorable recovery/resolution without persistent morbidity documented	21	Diagnostic vascular ultrasound used
03	03	3. Lateral geniculate artery pseudoaneurysm after arthroscopic medial meniscectomy.pdf	Bhamidipati	2024	USA	J Vasc Surg Cases Innov Tech	Lateral geniculate artery pseudoaneurysm after arthroscopic medial meniscectomy	Case report	No	32	M	No prior medical or surgical history reported	Meniscectomy	Uncomplicated arthroscopic medial meniscectomy	Specific arthroscopy/tourniquet/portal details not reported in full text	Lateral inferior genicular artery	Pulsatile mass at lateral knee	3 weeks postop	Duplex ultrasound; CTA; conventional angiography	Coil embolization of left LIGA	Symptoms resolved after coil embolization; discharged postoperative day 1 after follow-up duplex ultrasound		Full-text audit completed against available full text; unresolved fields marked Not reported	High		Meniscal procedure	Lateral/inferior genicular artery	Endovascular embolization/coiling	None separately reported	Favorable recovery/resolution without persistent morbidity documented	21	Diagnostic vascular ultrasound used; Angiography confirmatory/planning/treatment-associated or sequence mixed
04	04	4. Pseudoaneurysm of the superior lateral genicular artery following anterior cruciate ligament repair.pdf	Oversier	2018	Netherlands	Acta Chirurgica Belgica	Pseudoaneurysm of the superior lateral genicular artery following anterior cruciate ligament repair	Case report	No	25	M	Previously healthy; no fever at presentation; no comorbidity/coagulopathy reported	ACLR/ACL repair	Elective ACL ligamentoplasty using semitendinosus tendon autograft	ACL ligamentoplasty using autograft semitendinosus tendon; no peroperative complications; outside-in femoral targeting device implicated	Superior lateral genicular artery	Painful/growing knee swelling; pulsatile mass	3 weeks postop	Doppler ultrasound; CT angiography; angiography	Percutaneous thrombin injection/embolization reported	Recovered well; no sequelae noted		Full-text audit completed against available full text; unresolved fields marked Not reported	Moderate		ACL-related procedure	Superior lateral genicular artery	Ultrasound-guided thrombin injection	None separately reported	Favorable recovery/resolution without persistent morbidity documented	21	Diagnostic vascular ultrasound used; Angiography confirmatory/planning/treatment-associated or sequence mixed
05	05	5. Arthroscopy - 2009 - Kp - Popliteal Artery Pseudoaneurysm After Arthroscopic Cystectomy of a Popliteal Cyst.pdf	Venkatesh	2009	Korea	Arthroscopy	Popliteal artery pseudoaneurysm after arthroscopic cystectomy of a popliteal cyst	Case report	No	59	M	Not reported in full text	Other arthroscopic procedure	Arthroscopic cystectomy of popliteal cyst; associated medial meniscus symptoms	Arthroscopic cystectomy of popliteal cyst near popliteal fossa; additional portal/tourniquet details not reported in full text	Popliteal artery	Slow-growing painful popliteal mass; hypoesthesia/numbness later	2 months postop	Angiography	Open excision of pseudoaneurysm and popliteal artery pinpoint repair	30-month follow-up: pulses/sensation normal	Sensory symptoms before treatment	Full-text audit completed against available full text; unresolved fields marked Not reported	High		Synovectomy/debridement/irrigation/cyst procedure	Popliteal artery	Open surgical vascular treatment	None separately reported	Favorable recovery/resolution without persistent morbidity documented	60	Angiography served as primary/definitive vascular imaging
06	06	6. Ultrasound-Guided Thrombin Injection of Genicular Artery Pseudoaneurysm.pdf	Rachakonda	2015	USA	Annals of Vascular Surgery	Ultrasound-guided thrombin injection of genicular artery pseudoaneurysm	Case report	No	38	M	Not reported in full text	Diagnostic/therapeutic knee arthroscopy	Right knee arthroscopy	Not reported in full text	Inferior medial genicular artery	Medial knee pain/swelling; 2-cm pulsatile mass	1 month postop	Duplex ultrasound	Office-based ultrasound-guided thrombin injection	Resolution of active flow; no immediate/delayed complication at 6 weeks		Full-text audit completed against available full text; unresolved fields marked Not reported	High		Other diagnostic/therapeutic arthroscopy	Medial/inferior genicular artery	Ultrasound-guided thrombin injection	None separately reported	Favorable recovery/resolution without persistent morbidity documented	30	Diagnostic vascular ultrasound used
08	08	8. Pseudoaneurysm following hamstring tendon harvest in arthroscopic anterior cruciate ligament reconstruction_ a case report.pdf	Ho	2020	Taiwan	BMC Musculoskeletal Disorders	Pseudoaneurysm following hamstring tendon harvest in arthroscopic anterior cruciate ligament reconstruction: a case report	Case report	No	19	M	History of prolonged epistaxis; PT/INR/aPTT within reported reference ranges; no family bleeding/clotting disorder	ACLR	Arthroscopic ACLR using semitendinosus autograft/single-tunnel technique	Hamstring tendon harvest; semitendinosus autograft; single-tunnel technique	Saphenous branch of descending genicular artery	Large ecchymosis, focal swelling, tenderness around posteromedial knee; DVT/compartment syndrome ruled out	Postoperative day 6	Doppler ultrasound; angiography	Coil embolization	1-year follow-up improved functional outcome/good stability		Full-text audit completed against available full text; unresolved fields marked Not reported	High		ACL-related procedure	Descending genicular artery/branch	Endovascular embolization/coiling	None separately reported	Favorable recovery/resolution without persistent morbidity documented	6	Diagnostic vascular ultrasound used; Angiography confirmatory/planning/treatment-associated or sequence mixed
09	09A	9. Complete tibial nerve lesion secondary to postoperative popliteal pseudoaneurysm following anterior cruciate ligament arthroscopic reconstruction_ A series of two patients.pdf	Bonfim	2023	Austria	Surgical Neurology International	Complete tibial nerve lesion secondary to postoperative popliteal pseudoaneurysm following ACL arthroscopic reconstruction	Case report	No	34	M	Healthy	ACLR	Delayed arthroscopic ACL reconstruction	ACL reconstruction details not reported; arthroscopic reconstruction performed	Popliteal artery	Severe pain and paresthesia radiating to popliteal fossa/calf/foot; foot/ankle paralysis; initially DVT/compartment syndrome considered	Diagnosed after worsening by postop day 18	Doppler ultrasound to exclude DVT; angiography	Endovascular repair with seven covered stents; later nerve revision	Pain relieved but persistent motor/sensory deficit; nerve surgery required	Complete tibial/common peroneal nerve injury	Full-text audit completed against available full text; unresolved fields marked Not reported	High		ACL-related procedure	Popliteal artery	Covered stent/stent-graft	Nerve procedure	Persistent neurologic/functional morbidity or incomplete recovery	18	Diagnostic vascular ultrasound used; Angiography confirmatory/planning/treatment-associated or sequence mixed
09	09B	9. Complete tibial nerve lesion secondary to postoperative popliteal pseudoaneurysm following anterior cruciate ligament arthroscopic reconstruction_ A series of two patients.pdf	Bonfim	2023	Austria	Surgical Neurology International	Complete tibial nerve lesion secondary to postoperative popliteal pseudoaneurysm following ACL arthroscopic reconstruction	Case report	No	19	F	Healthy	ACLR	ACLR 3 years after skiing injury	ACL reconstruction details not reported; arthroscopic reconstruction performed	Popliteal artery	Continuous popliteal pain radiating to leg/foot; pulsating mass/blister; swelling; later paralysis	Diagnosed postop day 8	Initial Doppler negative; MR angiography; repeat Doppler	Endovascular repair; hematoma drainage; later tibial nerve reconstruction with sural nerve grafts	Peroneal nerve recovered; tibial nerve deficit persisted and improved after reconstruction	Tibial nerve rupture; peroneal/tibial neuropathy	Full-text audit completed against available full text; unresolved fields marked Not reported	High		ACL-related procedure	Popliteal artery	Covered stent/other endovascular repair	Hematoma/clot evacuation or drainage; Nerve procedure	Persistent neurologic/functional morbidity or incomplete recovery	8	Diagnostic vascular ultrasound used; Angiography confirmatory/planning/treatment-associated or sequence mixed
10	10A	10. pseudoaneurysm-after-therapeutic-knee-arthroscopy-imaging-findings.pdf	Hilborn	1994	Canada	AJR American Journal of Roentgenology	Pseudoaneurysm After Therapeutic Knee Arthroscopy: Imaging Findings	Case report	No	21	M	Not reported in full text	Other arthroscopic procedure	Therapeutic arthroscopy for osteochondritis dissecans; osteochondral fragment debridement/removal	Therapeutic knee arthroscopy; specific portal/tourniquet details not reported in full text	Popliteal artery	Knee swelling/discomfort; large firm popliteal mass with bruit	Symptoms ~1 week; diagnostic workup in ensuing weeks	Angiography; color Doppler ultrasound	Attempted ultrasound-guided compression failed; open excision/repair	Surgical repair; outcome not fully reported in the source report		Full-text audit completed against available full text; unresolved fields marked Not reported	High		Synovectomy/debridement/irrigation/cyst procedure	Popliteal artery	Open surgical vascular treatment	Attempted compression	Limited or short-term follow-up; definitive recovery not established		Diagnostic vascular ultrasound used; Angiography confirmatory/planning/treatment-associated or sequence mixed
10	10B	10. pseudoaneurysm-after-therapeutic-knee-arthroscopy-imaging-findings.pdf	Hilborn	1994	Canada	AJR American Journal of Roentgenology	Pseudoaneurysm After Therapeutic Knee Arthroscopy: Imaging Findings	Case report	No	49	M	Not reported in full text	Meniscectomy	Arthroscopy with torn meniscus resected	Inflow cannula site/vastus medialis region noted	Muscular branch of vastus medialis/superficial femoral branch	Medial superior knee swelling/induration; pulsatile mass	4 days postop symptoms; evaluated ~1 week postop	Selective femoral arteriography; color Doppler ultrasound	Attempted compression thrombosis failed/reperfused; coil embolization	Satisfactory resolution after coil embolization		Full-text audit completed against available full text; unresolved fields marked Not reported	High		Meniscal procedure	Superficial femoral/vastus branch	Endovascular embolization/coiling	Attempted compression	Favorable recovery/resolution without persistent morbidity documented	7	Diagnostic vascular ultrasound used; Angiography confirmatory/planning/treatment-associated or sequence mixed
11	11A	11. Sural artery pseudoaneurysms after knee arthroscopy_ Treatment with transcatheter embolization.pdf	Carlin	2001	USA	Journal of Vascular Surgery	Sural artery pseudoaneurysms after knee arthroscopy: treatment with transcatheter embolization	Case report	No	54	M	Not reported in full text	Diagnostic/therapeutic knee arthroscopy	Routine right knee arthroscopy for chronic knee pain	No intraoperative difficulties reported	Sural branch of popliteal artery	Calf tenderness, swelling, bruising; tender pulsatile popliteal mass; ecchymosis	1 week postop	Color duplex; retrograde arteriogram	Transcatheter embolization with thrombin/coils	Discharged day 1; duplex at 1 week showed no flow		Full-text audit completed against available full text; unresolved fields marked Not reported	High		Other diagnostic/therapeutic arthroscopy	Sural branch	Endovascular embolization/coiling	None separately reported	Favorable recovery/resolution without persistent morbidity documented	7	Diagnostic vascular ultrasound used; Angiography confirmatory/planning/treatment-associated or sequence mixed
11	11B	11. Sural artery pseudoaneurysms after knee arthroscopy_ Treatment with transcatheter embolization.pdf	Carlin	2001	USA	Journal of Vascular Surgery	Sural artery pseudoaneurysms after knee arthroscopy: treatment with transcatheter embolization	Case report	No	31	F	Not reported in full text	Diagnostic/therapeutic knee arthroscopy	Routine left knee arthroscopy for progressive knee pain	No intraoperative difficulties reported	Sural branch of popliteal artery with AVF	Severe calf pain; tender popliteal mass; calf swelling/ecchymosis	5 days postop	Color duplex; MRA; arteriogram	Subselective catheterization; coils and thrombin	Discharged day 1; duplex at 1 week showed no flow in PSA/AVF	Arteriovenous fistula	Full-text audit completed against available full text; unresolved fields marked Not reported	High		Other diagnostic/therapeutic arthroscopy	Sural branch	Endovascular embolization/coiling	None separately reported	Favorable recovery/resolution without persistent morbidity documented	5	Diagnostic vascular ultrasound used; Angiography confirmatory/planning/treatment-associated or sequence mixed
12	12	12. Pseudoaneurysm of the Popliteal Artery Leading to Foot Drop After Arthroscopic Anterior Cruciate Ligament Reconstruction_ A Rare Complication.pdf	Joshi	2019	Nepal	JBJS Case Connector	Pseudoaneurysm of the Popliteal Artery Leading to Foot Drop After Arthroscopic ACL Reconstruction	Case report	No	32	M	Serving soldier; no additional comorbidity/coagulopathy reported in full text	ACLR	Arthroscopic ACLR using hamstring autograft	Triple-looped semitendinosus hamstring autograft; anteromedial portal technique	Popliteal artery	Persistent posterior knee pain; at 4 weeks foot drop and intense posterior knee pain	4 weeks postop	Clinical examination plus vascular imaging reported; exact modality not reported	Surgical exploration, PSA excision, primary popliteal artery repair	Foot drop recovered at 3 months after second surgery	Foot drop/common peroneal palsy	Full-text audit completed against available full text; unresolved fields marked Not reported	High		ACL-related procedure	Popliteal artery	Open surgical vascular treatment	None separately reported	Favorable recovery/resolution without persistent morbidity documented	28	Role not separately classifiable
14	14	14. Pseudaneurysm of the superolateral genicular artery following an anterior cruciate ligament reconstruction.pdf	Glanz	2020	Switzerland	International Journal of Surgery Case Reports	Pseudoaneurysm of the superolateral genicular artery following ACL reconstruction	Case report	No	Not reported in full text	Not reported in full text	Not reported in full text	ACLR	ACLR with associated meniscal suture	Quadriceps tendon autograft; outside-in femoral tunnel technique; meniscal suture	Superolateral genicular artery	Pulsatile painless mass on external/lateral knee	19 days postop	CTA	Ultrasound-guided thrombin injection	No edema; full ROM; resolution without sequelae		Full-text audit completed against available full text; unresolved fields marked Not reported	Moderate		ACL-related procedure	Superior lateral genicular artery	Ultrasound-guided thrombin injection	None separately reported	Favorable recovery/resolution without persistent morbidity documented	19	Role not separately classifiable
15	15	15. Endovascular treatment of a pseudoaneurysm in the right inferomedial genicular artery after arthroscopic anterior cruciate ligament reconstruction_ a case report.pdf	Barrio Piqueras	2025	Spain	BJR Case Reports	Endovascular treatment of a pseudoaneurysm in the right inferomedial genicular artery after arthroscopic ACL reconstruction	Case report	No	47	M	Von Willebrand disease	ACLR	Right ACL reconstruction	Pseudoaneurysm near tibial tunnel screw; high origin anterior tibial artery	Articular branch of right inferomedial genicular artery arising from high-origin anterior tibial artery	Surgical wound dehiscence; suspected active bleeding; pulsatile swelling/bleeding	30 days postop	CT angiography; emergency angiography	Endovascular embolization with Squid Peri 18	Recovered well; no complications reported	Bleeding/wound dehiscence	Full-text audit completed against available full text; unresolved fields marked Not reported	High		ACL-related procedure	Medial/inferior genicular artery	Endovascular embolization/coiling	None separately reported	Favorable recovery/resolution without persistent morbidity documented	30	Angiography confirmatory/planning/treatment-associated or sequence mixed
16	16	16. Pseudoaneurysm of the Medial Superior Genicular Artery after Arthroscopic Partial Meniscectomy.pdf	Lee	2009	Korea	Clinics in Orthopedic Surgery	Pseudoaneurysm of the Medial Superior Genicular Artery after Arthroscopic Partial Meniscectomy	Case report	No	43	M	Not reported in full text	Meniscectomy	Arthroscopic partial medial meniscectomy	Standard anterolateral/anteromedial portals; basket forceps via anteromedial portal	Medial superior genicular artery	Painful effusion/hemarthrosis; pulsating mass at anteromedial portal	8 days hemarthrosis; persistent at 3 weeks postop	Arteriography	Transcatheter glue embolization	Pain-free at 6-week follow-up; no further vascular problem	Recurrent hemarthrosis	Full-text audit completed against available full text; unresolved fields marked Not reported	High		Meniscal procedure	Superior medial/middle genicular artery	Endovascular embolization/coiling	None separately reported	Favorable recovery/resolution without persistent morbidity documented	21	Angiography served as primary/definitive vascular imaging
17	17	17. Transarterial Embolization of an Inferior Genicular Artery Pseudoaneurysm with Arteriovenous Fistula after Arthroscopy.pdf	Puig	2007	Korea	Korean Journal of Radiology	Transarterial embolization of an inferior genicular artery pseudoaneurysm with arteriovenous fistula after arthroscopy	Case report	No	55	M	Not reported in full text	Meniscectomy	Arthroscopic meniscectomy	Not reported in full text	Lateral inferior genicular artery with AVF	Pseudoaneurysm with arteriovenous fistula after arthroscopy; presentation details not clearly reported in extractable full text	Not reported in full text	Angiography	Selective transarterial embolization	Successful minimally invasive treatment	Arteriovenous fistula	Full-text audit completed against available full text; unresolved fields marked Not reported	Moderate		Meniscal procedure	Lateral/inferior genicular artery	Endovascular embolization/coiling	None separately reported	Favorable recovery/resolution without persistent morbidity documented		Angiography served as primary/definitive vascular imaging
18	18	18. Arteriovenous fistula and pseudoaneurysm in a pediatric patient with persistent postoperative hemarthrosis_ A rare complication following arthroscopic repair of a bucket handle medial meniscal tear.pdf	Akan	2025	Turkey	Joint Diseases and Related Surgery	Arteriovenous fistula and pseudoaneurysm in a pediatric patient with persistent postoperative hemarthrosis: A rare complication following arthroscopic repair of a bucket handle medial meniscal tear	Case report	No	14	M	Not reported in full text	Meniscal repair	Arthroscopic repair of bucket-handle medial meniscal tear	Three all-inside meniscal sutures posteriorly/posteromedially and four inside-out meniscal sutures medially; discharged POD1	Superior medial geniculate artery AV fistula with subtotal occluded pseudoaneurysm	Persistent postoperative hemarthrosis	Postoperative day 7 presentation; vascular diagnosis after persistent hemarthrosis	MRI preoperatively; CT arteriogram for vascular diagnosis	Conservative management/close monitoring after cardiovascular surgery consultation	AV fistula closed and hemarthrosis resolved at 3 months; no vascular pathology at 2 years; Lysholm score 90	Persistent hemarthrosis; AVF	Full-text audit completed against available full text; unresolved fields marked Not reported	High		Meniscal procedure	Superior medial/middle genicular artery	Conservative observation	None separately reported	Favorable recovery/resolution without persistent morbidity documented		Angiography served as primary/definitive vascular imaging
19	19	19. An iatrogenic popliteal pseudoaneurysm masked under compartment syndrome of leg.pdf	Sajjad	2014	Ireland	Journal of Surgical Case Reports	An iatrogenic popliteal pseudoaneurysm masked under compartment syndrome of leg	Case report	No	60	M	Hypertension and hypothyroidism; taking candesartan and levothyroxine	Diagnostic/therapeutic knee arthroscopy	Knee arthroscopy before fasciotomy	Left knee arthroscopy and repair of meniscal tear; additional operative details not reported in full text	Popliteal artery	Pulsating mass behind knee; prior suspected compartment syndrome/fasciotomy failed to resolve symptoms	Swollen leg soon after surgery; pulsating mass recognized after several weeks	Ultrasound; CT angiogram	Emergency open repair via S-shaped popliteal incision; clot evacuation; arterial injury closed with short saphenous vein patch	Mobilizing by postoperative day 2; discharged within a week; 2-week follow-up wound healed with good peripheral pulses	Compartment syndrome mimic; fasciotomy prior to diagnosis	Full-text audit completed against available full text; unresolved fields marked Not reported	Moderate		Other diagnostic/therapeutic arthroscopy	Popliteal artery	Open surgical vascular treatment	Fasciotomy/decompression; Hematoma/clot evacuation or drainage	Favorable recovery/resolution without persistent morbidity documented		Diagnostic vascular ultrasound used
20	20	20. Volume rendered three-dimensional CT angiography for the diagnosis of arteriovenous fistula and pseudoaneurysm of the popliteal artery following knee arthroscopy.pdf	Joshi	2014	UK	BMJ Case Reports	Volume rendered 3D CT angiography for AVF and popliteal artery pseudoaneurysm following knee arthroscopy	Case report	No	24	F	Not reported in full text	Diagnostic arthroscopy	Diagnostic knee arthroscopy	Not reported in full text	Popliteal artery with AVF	Swollen leg and pulsatile lump in popliteal fossa	3 days postop	CT arteriogram with volume-rendered 3D CTA	Open repair eventually performed (stent considered but avoided)	Resolution of venous hypertension and preservation of distal arterial perfusion	Arteriovenous fistula; venous hypertension	Full-text audit completed against available full text; unresolved fields marked Not reported	High		Other diagnostic/therapeutic arthroscopy	Popliteal artery	Open surgical vascular treatment	None separately reported	Favorable recovery/resolution without persistent morbidity documented	3	Angiography confirmatory/planning/treatment-associated or sequence mixed
21	21	21. Pseudoaneurysm with Arteriovenous Fistula after Arthroscopic Procedure_ A Rare Complication of Arthroscopy.pdf	Jin	2015	Korea	Korean Journal of Thoracic and Cardiovascular Surgery	Pseudoaneurysm with arteriovenous fistula after arthroscopic procedure: A rare complication of arthroscopy	Case report	No	23	M	No significant prior medical history	Cruciate ligament arthroscopy	Arthroscopic surgery for cruciate ligament injury	Arthroscopic surgery for cruciate ligament injury; additional surgical details not reported in full text	Descending genicular artery pseudoaneurysm with fistula to superficial vein of right vastus medialis	Growing 6 x 4 cm pulsatile oval mass around prior superomedial surgical wound with bruit	5 months postop	CT angiography; Doppler/USG/angiography discussed	Pseudoaneurysm resection and AVF ligation	Postoperative course unremarkable; discharged on postoperative day 7	Arteriovenous fistula	Full-text audit completed against available full text; unresolved fields marked Not reported	Moderate		PCL/combined cruciate procedure	Descending genicular artery/branch	Open surgical vascular treatment	None separately reported	Limited or short-term follow-up; definitive recovery not established	150	Ultrasound not counted as primary diagnostic vascular ultrasound; Angiography confirmatory/planning/treatment-associated or sequence mixed
22	22	22. Pseudoaneurysm_of_the_Descending_Genicular_Artery.pdf	Tozzi	1996	Italy	Journal of Trauma	Pseudoaneurysm of the Descending Genicular Artery after Arthroscopic Meniscectomy	Case report	No	39	M	Not reported in full text	Meniscectomy	Medial arthroscopic meniscectomy	Tourniquet used; portal details not reported in full text	Descending genicular artery	Pulsatile mass on medial upper side of knee above joint line after medial meniscectomy	8 days postop	CT scan; angiography	Surgical resection	Complete recovery/return to normal daily life		Full-text audit completed against available full text; unresolved fields marked Not reported	High		Meniscal procedure	Descending genicular artery/branch	Open surgical vascular treatment	None separately reported	Favorable recovery/resolution without persistent morbidity documented	8	Angiography confirmatory/planning/treatment-associated or sequence mixed
23	23	23. Arthroscopy - 2004 - Janssen - Pseudoaneurysm of the popliteal artery after anterior cruciate ligament reconstruction with.pdf	Janssen	2004	Netherlands	Arthroscopy	Pseudoaneurysm of the popliteal artery after ACL reconstruction with bicortical tibial screw fixation	Case report	No	24	M	Not reported in full text	ACLR	Arthroscopic ACLR using quadruple hamstring graft	Bone Mulch Screw femoral fixation; bicortical WasherLoc tibial fixation	Infragenicular popliteal artery	Progressive pain in popliteal fossa; painful pulsatile swelling	12 days postop	CT angiography; angiography	Open surgical exploration/repair of infragenicular popliteal artery pseudoaneurysm	Distal pulses intact after treatment; follow-up details not otherwise reported in source report	Hardware-related injury near bicortical tibial screw	Full-text audit completed against available full text; unresolved fields marked Not reported	High		ACL-related procedure	Popliteal artery	Open surgical vascular treatment	None separately reported	Limited or short-term follow-up; definitive recovery not established	12	Angiography confirmatory/planning/treatment-associated or sequence mixed
24	24	24. Arthroscopy - 2011 - Mello - Pseudoaneurysm of the Medial Inferior Genicular Artery After Anterior Cruciate Ligament.pdf	Mello	2011	Brazil	Arthroscopy	Pseudoaneurysm of the Medial Inferior Genicular Artery After ACL Reconstruction	Case report	No	23	M	Not reported in full text	ACLR	ACL reconstruction using central third patellar tendon graft	Patellar tendon graft; absorbable interference screw fixation	Medial inferior genicular artery	Repeated voluminous hemarthrosis during physiotherapy	Second postoperative week/early postop	Magnetic resonance angiography; angiography	Transluminal/selective embolization	Good outcome; no recurrence reported at follow-up	Recurrent hemarthrosis	Full-text audit completed against available full text; unresolved fields marked Not reported	High		ACL-related procedure	Medial/inferior genicular artery	Endovascular embolization/coiling	None separately reported	Favorable recovery/resolution without persistent morbidity documented	14	Angiography confirmatory/planning/treatment-associated or sequence mixed
25	25	25. Popliteal artery pseudoaneurysm following arthroscopic lateral meniscus root repair.pdf	Iqbal	2025	India	Future Health	Popliteal artery pseudoaneurysm following arthroscopic lateral meniscus root repair	Case report	No	31	M	Athlete; no additional comorbidity/coagulopathy reported in full text	Meniscal repair/root repair + ACLR	Arthroscopic lateral meniscus root repair; ACLR also performed	FAST-FIX meniscus sutures; transtibial pullout root repair; semitendinosus ACL graft with EndoButton/Bioscrew	Popliteal artery	Postop pain/swelling; normal distal circulation	4th postop day symptoms; CTA early postop	CT angiogram; Doppler/CTA follow-up	Conservative observation/surveillance; rehabilitation de-escalated	Complete resolution on 2-month CT angiogram; functional/pain-free limb		Full-text audit completed against available full text; unresolved fields marked Not reported	High		Combined ACL and meniscal procedure	Popliteal artery	Conservative observation	None separately reported	Favorable recovery/resolution without persistent morbidity documented	4	Ultrasound not counted as primary diagnostic vascular ultrasound
26	26	26. Limb#U2010Threatening Complication After Knee Arthroscopy.pdf	Smoter	2025	Slovakia	Bratislava Medical Journal	Limb-Threatening Complication After Knee Arthroscopy	Case report + literature review	Extracted only the new original case; ignore review table cases	59	F	No prior medical history; nonsmoker; no known coagulopathy	Meniscectomy/debridement	Elective therapeutic left knee arthroscopy with meniscus resection in posterior segment	Not reported in full text	Popliteal artery	Left popliteal pain; progressive swelling; aspiration of suspected Baker cyst	28 days postop aspiration; diagnosis thereafter	Ultrasound guidance initially; CT angiography	Open direct repair through lazy S incision with small saphenous vein patch angioplasty	Post-procedure follow-up showed symptom resolution and patent runoff	Limb-threatening presentation/rupture risk	Full-text audit completed against available full text; unresolved fields marked Not reported	High		Meniscal procedure	Popliteal artery	Open surgical vascular treatment	None separately reported	Favorable recovery/resolution without persistent morbidity documented	28	Ultrasound not counted as primary diagnostic vascular ultrasound; Angiography confirmatory/planning/treatment-associated or sequence mixed
28	28	28. Pseudoaneurysm of the superolateral genicular artery following ACL outside-in reconstruction.pdf	Castroviejo	2024	Switzerland	BMJ Case Reports	Pseudoaneurysm of the superolateral genicular artery following ACL outside-in reconstruction	Case report	No	20s	F	Unremarkable history	ACLR	ACLR plus anterolateral ligament reconstruction using outside-in drilling technique	ACL plus anterolateral ligament reconstruction using outside-in drilling; tourniquet deflation discussed; graft/fixation details not otherwise reported in source report	Superolateral genicular artery	Painful pulsatile mass on lateral femoral condyle	4 weeks postop	CT angiography; imaging confirmation	Thrombin injections twice	Reduced size and minimal pain at follow-up		Full-text audit completed against available full text; unresolved fields marked Not reported	High		ACL-related procedure	Superior lateral genicular artery	Ultrasound-guided thrombin injection	None separately reported	Limited or short-term follow-up; definitive recovery not established	28	Angiography confirmatory/planning/treatment-associated or sequence mixed
29	29	29. Delayed Pseudoaneurysm of the Popliteal Artery Following ACL Reconstruction.pdf	Ambrosia	2015	USA	Orthopedics	Delayed Pseudoaneurysm of the Popliteal Artery Following ACL Reconstruction	Case report	No	18	F	Soccer player; no additional comorbidity/coagulopathy reported in full text	ACLR	ACL reconstruction with ipsilateral autologous hamstring graft plus medial meniscal repair	Hamstring autograft; meniscal repair; popliteal artery spasm treated intraop/postop with balloon angioplasty	Popliteal artery	Foot paresthesias; pseudoaneurysm after prior popliteal spasm	7 weeks postop	Vascular imaging diagnosed 3.5 x 3.1 x 1.3 cm pseudoaneurysm; exact modality not reported	Vascular intervention performed; exact treatment not reported	Not reported in full text	Foot paresthesias; prior popliteal artery spasm	Full-text audit completed against available full text; unresolved fields marked Not reported	Moderate		ACL-related procedure	Popliteal artery	Vascular treatment, approach not reported	None separately reported	Outcome not reported	49	Role not separately classifiable
33	33	33. Pseudo-aneurysm of the popliteal artery following an arthroscopic meniscectomy-report of a pediatric case.pdf	Ben Romdhane	1991	France	Pediatric Radiology	Pseudo-aneurysm of the popliteal artery following an arthroscopic meniscectomy: report of a pediatric case	Case report	No	8	M	Not reported in full text	Meniscectomy	Arthroscopic meniscectomy for torn discoid lateral meniscus	Not reported in full text	Popliteal artery	Hemarthrosis; painful/warm/firm non-pulsatile popliteal mass with systolic murmur	Hemarthrosis 1 week; mass 1 month postop	Ultrasound; angiography	Resection and end-to-end anastomosis	Surgical repair performed; detailed follow-up outcome not reported in full text	Pediatric case; hemarthrosis	Full-text audit completed against available full text; unresolved fields marked Not reported	High		Meniscal procedure	Popliteal artery	Open surgical vascular treatment	None separately reported	Outcome not reported	30	Diagnostic vascular ultrasound used; Angiography confirmatory/planning/treatment-associated or sequence mixed
34	34	34. Delayed compartment syndrome of leg and foot due to rupture of popliteal artery pseudoaneurysm following posterior cruciate ligament reconstruction.pdf	Shahrulazua	2014	Malaysia	BMJ Case Reports	Delayed compartment syndrome of leg and foot due to rupture of popliteal artery pseudoaneurysm following PCL reconstruction	Case report	No	28	M	Prior severe knee/leg trauma with femur/tibia/fibula fractures; implants removed	PCL reconstruction	Arthroscopic single-bundle transtibial PCL reconstruction	Autologous hamstrings graft; transtibial PCL technique	Popliteal artery	Increasing leg/foot pain; compartment syndrome signs; paresthesia/reduced pulses	9 days postop	CTA	Exploration with hematoma evacuation and restoration of distal blood flow; fasciotomies of leg and foot compartments	Recovered without further complication after rehab (as reported)	Ruptured PSA; delayed compartment syndrome of leg/foot	Full-text audit completed against available full text; unresolved fields marked Not reported	High		PCL/combined cruciate procedure	Popliteal artery	Open surgical vascular treatment	Fasciotomy/decompression; Hematoma/clot evacuation or drainage	Favorable recovery/resolution without persistent morbidity documented	9	Role not separately classifiable
35	35	35. Delayed diagnosis of a pseudoaneurysm with recurrent hemarthrosis of the knee joint.pdf	Becher	2008	Germany	Knee Surgery Sports Traumatology Arthroscopy	Delayed diagnosis of a pseudoaneurysm with recurrent hemarthrosis of the knee joint	Case report	No	47	M	Multiple previous arthroscopic procedures; hyaluronic acid injections; prior high tibial osteotomy/abrasion arthroplasty noted	Synovectomy/arthroscopy	Three arthroscopic procedures/multiple punctures; first arthroscopic synovectomy for suspected infection	Multiple punctures/arthroscopies; causality less clean	Superior middle genicular artery	Massive swelling/severe pain; recurrent hemarthrosis over 3 months	Delayed over 3 months	Color duplex ultrasound; angiography	Selective angiographic embolization	Successful treatment	Recurrent hemarthrosis; delayed diagnosis	Full-text audit completed against available full text; unresolved fields marked Not reported	High	Include but flag causality because multiple punctures/arthroscopies occurred before diagnosis	Synovectomy/debridement/irrigation/cyst procedure	Superior medial/middle genicular artery	Endovascular embolization/coiling	None separately reported	Favorable recovery/resolution without persistent morbidity documented		Diagnostic vascular ultrasound used; Angiography confirmatory/planning/treatment-associated or sequence mixed
36	36	36. Endovascular repair of popliteal artery pseudoaneurysm with arteriovenous fistula after knee arthroscopy-case report and literature review.pdf	Alserr	2014	Greece	Vascular and Endovascular Surgery	Endovascular repair of popliteal artery pseudoaneurysm with arteriovenous fistula after knee arthroscopy: case report and literature review	Case report + literature review	Extracted only new original case; ignore review table cases	53	F	Not reported in full text	Meniscectomy	Arthroscopic meniscectomy	Not reported in full text	Popliteal artery with AVF	Limb edema, pain, bruit	1 year postop	Duplex ultrasound and angiography/CTA used for diagnosis and endovascular planning	Covered stent deployment	Asymptomatic at 6 months; patent stent; AVF occluded	Arteriovenous fistula	Full-text audit completed against available full text; unresolved fields marked Not reported	High		Meniscal procedure	Popliteal artery	Covered stent/stent-graft	None separately reported	Favorable recovery/resolution without persistent morbidity documented	365	Diagnostic vascular ultrasound used; Angiography confirmatory/planning/treatment-associated or sequence mixed
37	37	37. Clinical image- popliteal artery pseudoaneurysm following arthroscopic synovectomy.pdf	Lee	2007	South Korea	Arthritis & Rheumatism	Clinical image: popliteal artery pseudoaneurysm following arthroscopic synovectomy	Case report	No	72	F	Chronic synovitis; no additional comorbidity/coagulopathy reported in full text	Synovectomy	Arthroscopic synovectomy of both knees for chronic synovitis	Arthroscopic synovectomy of both knees for chronic synovitis; additional surgical details not reported	Popliteal artery	Swelling of popliteal fossa/lower leg; edema; inability to flex; pulsating mass with bruit; weak dorsalis pedis pulse	11 days postop	Doppler ultrasound; 3D CT angiography	Vascular surgery with complete pseudoaneurysm resection and autogenous saphenous vein graft interposition	Uneventful postoperative course; good distal dorsalis pedis pulses at discharge	Positive Homans sign/DVT-like	Full-text audit completed against available full text; unresolved fields marked Not reported	Moderate		Synovectomy/debridement/irrigation/cyst procedure	Popliteal artery	Open surgical vascular treatment	None separately reported	Limited or short-term follow-up; definitive recovery not established	11	Diagnostic vascular ultrasound used; Angiography confirmatory/planning/treatment-associated or sequence mixed
38	38	38. Popliteal pseudoaneurysm and arteriovenous shunt following arthroscopic meniscectomy- case report..pdf	Vassallo	1989	Germany	Cardiovascular and Interventional Radiology	Popliteal pseudoaneurysm and arteriovenous shunt following arthroscopic meniscectomy	Case report	No	55	M	Prior meniscectomy 8 years earlier; valgus tibial osteotomy 3 years earlier	Meniscectomy	Arthroscopic resection of remnants of posterior horn of medial meniscus	Repeat arthroscopy with arthroscopic resection of posterior horn medial meniscus remnants; tourniquet masking discussed, but operative tourniquet use not explicitly stated	Popliteal artery with arteriovenous shunt to popliteal vein	Increasing popliteal fossa pain/swelling over 7 days; cold limb; absent peripheral pulses; pulsatile popliteal mass	7 days postop	Digital subtraction angiography	Excision of aneurysm and fistula with end-to-end anastomoses of popliteal artery and vein	Postoperative period uneventful	Arteriovenous shunt	Full-text audit completed against available full text; unresolved fields marked Not reported	Moderate		Meniscal procedure	Popliteal artery	Open surgical vascular treatment	None separately reported	Limited or short-term follow-up; definitive recovery not established	7	Angiography served as primary/definitive vascular imaging
39	39	39. Endovascular repair of a pseudoaneurysm of the popliteal artery following arthroscopic knee surgery-case report and review of literature.pdf	AbuRahma	2013	USA	Vascular	Endovascular repair of a pseudoaneurysm of the popliteal artery following arthroscopic knee surgery	Case report + literature review	Extracted only new original case; ignore review table cases	52	M	Denied chest pain/shortness of breath; no comorbidity/coagulopathy reported in full text	Meniscectomy	Arthroscopic knee surgery/repair for torn meniscus	Arthroscopic repair for torn meniscus; additional operative details not reported in full text	Popliteal artery	Sudden left lower extremity pain radiating to calf/foot	4 days postop	Bilateral lower-extremity duplex ultrasound; arterial duplex; intravascular ultrasound; arteriography	Endovascular covered stent repair	Postoperative duplex showed patent stent graft; satisfactory sealing		Full-text audit completed against available full text; unresolved fields marked Not reported	High		Meniscal procedure	Popliteal artery	Covered stent/stent-graft	None separately reported	Favorable recovery/resolution without persistent morbidity documented	4	Diagnostic vascular ultrasound used; Angiography confirmatory/planning/treatment-associated or sequence mixed
40	40	40. Pseudoaneurysm of the articular branch of the descending genicular artery following double-bundle anterior cruciate ligament reconstruction.pdf	Tsubosaka	2017	Japan	Knee Surgery Sports Traumatology Arthroscopy	Pseudoaneurysm of the articular branch of the descending genicular artery following double-bundle anterior cruciate ligament reconstruction	Case report	No	18	M	Not reported in full text	ACLR	Anatomic double-bundle ACL reconstruction	Semitendinosus graft; far anteromedial portal technique	Articular branch of descending genicular artery	Pulsatile medial knee swelling on second postop day	2 days postop	Contrast CT; Doppler ultrasonography	Microcatheter embolization	No additional complications reported		Full-text audit completed against available full text; unresolved fields marked Not reported	High		ACL-related procedure	Descending genicular artery/branch	Endovascular embolization/coiling	None separately reported	Limited or short-term follow-up; definitive recovery not established	2	Diagnostic vascular ultrasound used
41	41	41. Pseudoaneurysm after arthroscopic procedure in the knee.pdf	Stieven Filho	2015	Brazil	Revista Brasileira de Ortopedia	Pseudoaneurysm after arthroscopic procedure in the knee	Case report + literature review	Extracted only new original case; do not extract review table cases	17	M	Not reported in full text	ACLR	Arthroscopic ACL reconstruction after handball injury	Revision ACL reconstruction using quadriceps tendon graft; extensive repair of femoral intercondylar area	Inferomedial genicular artery	Joint effusion and painful pulsatile inferomedial knee mass	Postoperative day 1	Echo Doppler; arteriography	Embolization by vascular surgery	Good recovery without intercurrences		Full-text audit completed against available full text; unresolved fields marked Not reported	High		ACL-related procedure	Medial/inferior genicular artery	Endovascular embolization/coiling	None separately reported	Favorable recovery/resolution without persistent morbidity documented	1	Diagnostic vascular ultrasound used; Angiography confirmatory/planning/treatment-associated or sequence mixed
42	42	42. Pseudoaneurysm complicating knee arthroscopy A case report.pdf	Audenaert	2003	Belgium	Acta Orthopaedica Belgica	Pseudoaneurysm complicating knee arthroscopy: A case report	Case report	No	66	F	Not reported in full text	Meniscectomy	Partial arthroscopic medial meniscectomy	Partial arthroscopic medial meniscectomy; pneumatic thigh tourniquet used during surgical exploration; arthroscopy portal details not reported	Narrow-necked filiform muscular arterial branch to gastrocnemius muscle	Massive edema, ecchymosis, calf pain; initially diagnosed as DVT	3 weeks postop	CT angiography; angiography	Pseudoaneurysm resection with distal branch ligation and hematoma drainage	Good distal flow after surgery; rehabilitation uneventful; discharged two days after surgery	DVT-like presentation; calf hematoma	Full-text audit completed against available full text; unresolved fields marked Not reported	High		Meniscal procedure	Other branch	Open surgical vascular treatment	Hematoma/clot evacuation or drainage	Favorable recovery/resolution without persistent morbidity documented	21	Angiography confirmatory/planning/treatment-associated or sequence mixed
43	43	43. Pseudoaneurysm of the medial inferior genicular artery following anterior cruciate ligament reconstruction.pdf	Evans	2000	UK	Annals of the Royal College of Surgeons of England	Pseudoaneurysm of the medial inferior genicular artery following ACL reconstruction	Case report	No	30	M	Previously fit	ACLR	ACL reconstruction using central third patellar tendon graft	Patellar tendon graft fixed with tibial/femoral interference screws	Medial inferior genicular artery with AVF/late emptying into saphenous vein	2-cm pulsatile medial knee swelling; Doppler murmur	5 weeks postop presentation with swelling; vascular diagnosis after clinical/Doppler evaluation	Doppler; angiography	Open surgery with aneurysm resection and reverse long saphenous vein graft	Good functional result reported	Arteriovenous fistula/venous communication	Full-text audit completed against available full text; unresolved fields marked Not reported	High		ACL-related procedure	Medial/inferior genicular artery	Open surgical vascular treatment	None separately reported	Favorable recovery/resolution without persistent morbidity documented	35	Diagnostic vascular ultrasound used; Angiography confirmatory/planning/treatment-associated or sequence mixed
44	44	44. Minimally invasive treatment of portal pseudoaneurysm in a haemophiliac after knee arthroscopy.pdf	Leung	2020	Hong Kong	Journal of Orthopaedic Surgery	Minimally invasive treatment of portal pseudoaneurysm in a haemophiliac after knee arthroscopy	Case report	No	23	M	Haemophilia A; factor VIII replacement required	ACLR	Arthroscopic-assisted ACL reconstruction after knee sprain	Single-bundle hamstring graft; anteromedial portal pseudoaneurysm	Branches of medial inferior geniculate artery/portal pseudoaneurysm	Swelling over anteromedial portal scar; recurrent after first treatment	24 weeks postop swelling over anteromedial portal scar; residual/recurrent flow treated with repeat injection 6 weeks later	Ultrasound	Ultrasound-guided thrombin injection; repeat injection at 6 weeks	Clinical swelling resolved; no flow on follow-up ultrasound	Coagulopathy/haemophilia; recurrence/residual slow flow after first injection	Full-text audit completed against available full text; unresolved fields marked Not reported	High		ACL-related procedure	Medial/inferior genicular artery	Ultrasound-guided thrombin injection	None separately reported	Favorable recovery/resolution without persistent morbidity documented	168	Diagnostic vascular ultrasound used
45	45	45. Pseudoaneurysm in the anterior tibial artery after arthroscopic anterior cruciate ligament repair_ A case report.pdf	Simmons	2022	USA	Trauma Case Reports	Pseudoaneurysm in the anterior tibial artery after arthroscopic ACL repair	Case report	No	32	F	Not reported in full text	ACLR/ACL repair	Arthroscopically assisted ACL reconstruction/repair with meniscus repair	Semitendinosus allograft quadrupled with attachable buttons on tibial/femoral sides; tibial guide 55°; medial meniscus all-inside fixation with FiberLink sutures	Anterior tibial artery	Palpable fluctuance/firm painful mass; new foot drop	Fluctuance 1 week; foot drop ~6 weeks postop	Bedside duplex ultrasound; CT angiography	Vascular surgery evacuated hematoma and repaired pseudoaneurysm	Improved pain/dysesthesias and toe motion 2 weeks after repair; by 7 months knee pain resolved and foot drop improved, no AFO needed though slight limp persisted	Foot drop/peroneal nerve compression	Full-text audit completed against available full text; unresolved fields marked Not reported	Moderate		ACL-related procedure	Anterior tibial artery	Vascular treatment, approach not reported	None separately reported	Persistent neurologic/functional morbidity or incomplete recovery		Diagnostic vascular ultrasound used; Angiography confirmatory/planning/treatment-associated or sequence mixed
46	46	46. Pseudoaneurysm of the Superior Lateral Genicular Artery of the Knee Following Arthroscopic Irrigation and Debridement and Review of Literature .pdf	Gliatis	2023	Greece	Indian Journal of Orthopaedics	Pseudoaneurysm of the Superior Lateral Genicular Artery Following Arthroscopic Irrigation and Debridement	Case report + literature review	Extracted only new original case; ignore review table cases	16	M	Septic arthritis after prior meniscal repair; no comorbidities	Irrigation/debridement/synovectomy	Arthroscopic irrigation and debridement for septic arthritis after prior lateral meniscus repair	Prior arthroscopic lateral meniscus repair 2 years before; active bleeding from lateral portal	Superior lateral genicular artery (also bleeding from superior/inferior lateral branches noted)	Right knee pain/swelling; active pulsatile bleeding from lateral portal	Returned 10 days after discharge with recurrent pain/swelling and active pulsatile bleeding from lateral portal	CTA	Intravascular embolization	No clinical/lab signs of infection during follow-up	Bleeding; septic arthritis context	Full-text audit completed against available full text; unresolved fields marked Not reported	High		Synovectomy/debridement/irrigation/cyst procedure	Superior lateral genicular artery	Endovascular embolization/coiling	None separately reported	Limited or short-term follow-up; definitive recovery not established	10	Role not separately classifiable
47	47	47. Popliteal pseudoaneurysm and arteriovenous fistula after arthroscopic anterior and posterior cruciate ligament reconstruction_ A case reportmain.pdf	Xu	2017	China	International Journal of Surgery Case Reports	Popliteal pseudoaneurysm and AVF after arthroscopic ACL and PCL reconstruction	Case report	No	57	M	Not reported in full text	ACLR/PCL reconstruction	Arthroscopic ACL and PCL reconstruction	Semitendinosus graft from both limbs	Popliteal artery with AVF	Pain and swelling around left lower extremity	6 months postop	Doppler ultrasound; CTA	Vascular repair performed for popliteal pseudoaneurysm/AVF; exact technique not reported	Not reported in full text	Arteriovenous fistula	Full-text audit completed against available full text; unresolved fields marked Not reported	Moderate		PCL/combined cruciate procedure	Popliteal artery	Vascular treatment, approach not reported	None separately reported	Outcome not reported	180	Diagnostic vascular ultrasound used
48	48	48. Popliteal Artery Pseudoaneurysm after Anterior Cruciate Ligament Re-Revision Using a Rigidfix Cross Pin.pdf	Lee	2014	Korea	Knee Surgery & Related Research	Popliteal Artery Pseudoaneurysm after ACL Re-Revision Using a RigidFix Cross Pin	Case report	No	Not reported	Not reported	Prior ACL reconstruction/revision; re-rupture history	ACLR revision/re-revision	ACL re-revision using transtibial femoral tunnel technique	RigidFix cross pin; previous Achilles tendon allografts	Popliteal artery	Popliteal fossa pain starting night of surgery; swelling; common peroneal nerve palsy	2 days postop worsening; 6 weeks after surgery at referral	MRI and CT angiography/angiography reported in case evaluation	Vascular treatment performed; exact technique not reported	Not reported	Common peroneal nerve palsy; hardware-related concern	Full-text audit completed against available full text; unresolved fields marked Not reported	Medium		ACL-related procedure	Popliteal artery	Vascular treatment, approach not reported	None separately reported	Outcome not reported		Angiography confirmatory/planning/treatment-associated or sequence mixed
49	49	49. kwok-wai-et-al-2014-pseudoaneurysm-of-the-popliteal-artery-after-anterior-cruciate-ligament-reconstruction-surgery-a.pdf	Tam	2014	Hong Kong	Journal of Orthopaedics, Trauma and Rehabilitation	Pseudoaneurysm of the Popliteal Artery After ACL Reconstruction Surgery	Case report + literature review	Extracted only new original case; ignore review table cases	28	M	Not reported in full text	ACLR	ACL reconstruction	ACL reconstruction; additional graft/fixation/tourniquet details not reported in full text	Popliteal artery	Painful calf swelling; compartment syndrome	8 days postop	Doppler ultrasound; surgical findings	Fasciotomy with hematoma evacuation followed by MRI/CT angiogram confirmation and open surgical repair of punctured popliteal artery/pseudoaneurysm	Not reported in full text	Compartment syndrome	Full-text audit completed against available full text; unresolved fields marked Not reported	High		ACL-related procedure	Popliteal artery	Open surgical vascular treatment	Fasciotomy/decompression; Hematoma/clot evacuation or drainage	Outcome not reported	8	Diagnostic vascular ultrasound used
50	50	50. Inferior medial geniculate artery pseudoaneurysm complicating arthroscopic partial meniscectomy.pdf	Ellis	2018	Australia	ANZ Journal of Surgery	Inferior medial geniculate artery pseudoaneurysm complicating arthroscopic partial meniscectomy	Case report	No	51	M	Mild haemophilia A	Meniscectomy	Arthroscopic partial medial meniscectomy	Not reported in full text	Inferior medial geniculate artery	Large protruberant soft tissue mass; discomfort walking/squatting	Mass began week after procedure; presented 4 months postop	Doppler ultrasound	Ultrasound-guided thrombin injection	Returned to usual treating surgeon; immediate success noted	Coagulopathy/haemophilia	Full-text audit completed against available full text; unresolved fields marked Not reported	High		Meniscal procedure	Medial/inferior genicular artery	Ultrasound-guided thrombin injection	None separately reported	Limited or short-term follow-up; definitive recovery not established	120	Diagnostic vascular ultrasound used
51	51	51. Pseudoaneurysm of the popliteal artery following arthroscopic meniscectomy.pdf	Hussein	1998	USA	Bulletin Hospital for Joint Diseases	Pseudoaneurysm of the popliteal artery following arthroscopic meniscectomy	Case report	No	40	F	Diabetes type A reported	Meniscectomy	Arthroscopic meniscectomy for posterior horn medial meniscus tear	Not reported in full text	Popliteal artery	Popliteal pseudoaneurysm diagnosed after arthroscopic meniscectomy; detailed presentation not reported in source report	Not reported in full text	Ultrasound; angiography	Reversed saphenous vein graft repair	Treated successfully		Full-text audit completed against available full text; unresolved fields marked Not reported	High		Meniscal procedure	Popliteal artery	Open surgical vascular treatment	None separately reported	Favorable recovery/resolution without persistent morbidity documented		Diagnostic vascular ultrasound used; Angiography confirmatory/planning/treatment-associated or sequence mixed
52	52	52. Arthroscopy - 2001 - Kiss - Postoperative aneurysm of the popliteal artery after arthroscopic meniscectomy.pdf	Kiss	2001	Austria	Arthroscopy	Postoperative Aneurysm of the Popliteal Artery After Arthroscopic Meniscectomy	Case report	No	14	F	Not reported in full text	Meniscectomy	Arthroscopic subtotal meniscectomy for bucket-handle lateral meniscus tear	General anesthesia and distal femoral tourniquet; arthroscopic subtotal meniscectomy	Popliteal artery	Reduced sensory/motor response in forefoot on postop day 2; later vascular diagnosis	Postoperative day 2 neurologic symptoms; CT diagnosis after persistent pain following 1-week hematoma aspiration	Three-dimensional computed tomography	Revision surgery with saphena magna patch reconstruction	1-year follow-up: excellent ROM/function, normal quality of life; pain-free/full ROM	Neurologic symptoms/forefoot sensory-motor deficit	Full-text audit completed against available full text; unresolved fields marked Not reported	High		Meniscal procedure	Popliteal artery	Open surgical vascular treatment	None separately reported	Favorable recovery/resolution without persistent morbidity documented		Role not separately classifiable
53	53	53. Aneurysm after arthroscopy.pdf	Manning	1987	UK	Journal of Bone and Joint Surgery British Volume	Aneurysm after arthroscopy	Case report	No	23	M	Not reported in full text	Diagnostic arthroscopy	Diagnostic arthroscopy after football injury	Pneumatic thigh tourniquet	Inferior lateral geniculate artery	Recurrent hemarthroses; pulsatile swellings with bruit	2 weeks postop recognized pulsatile swelling; readmitted 6 weeks later	Clinical diagnosis; no vascular imaging reported before exploration	Open excision of false aneurysm and double ligation of feeding inferior lateral geniculate artery	Uneventful recovery; later required ACL prosthetic replacement	Recurrent hemarthrosis	Full-text audit completed against available full text; unresolved fields marked Not reported	Moderate		Other diagnostic/therapeutic arthroscopy	Lateral/inferior genicular artery	Open surgical vascular treatment	None separately reported	Favorable recovery/resolution without persistent morbidity documented	14	Role not separately classifiable
55	55	55. Arthroscopy - 1997 - Sarrosa - Pseudoaneurysm as a complication of knee arthroscopy.pdf	Sarrosa	1997	Canada	Arthroscopy	Pseudoaneurysm as a complication of knee arthroscopy	Case report	No	32	M	Not reported in full text	Meniscectomy	Arthroscopy after water-skiing knee injury; meniscus trimmed	Pneumatic thigh tourniquet	Inferior medial geniculate artery	Pulsatile swelling/mass; clinically suspected pseudoaneurysm	Not reported in full text	Peripheral angiography; Doppler/MRI discussed	Coil embolization	No more extravasation after embolization		Full-text audit completed against available full text; unresolved fields marked Not reported	High		Meniscal procedure	Medial/inferior genicular artery	Endovascular embolization/coiling	None separately reported	Favorable recovery/resolution without persistent morbidity documented		Ultrasound not counted as primary diagnostic vascular ultrasound; Angiography confirmatory/planning/treatment-associated or sequence mixed
56	56	56. Arthroscopy - 2001 - Mullen - Popliteal pseudoaneurysm and arteriovenous fistula after arthroscopic meniscectomy.pdf	Mullen	2001	USA	Arthroscopy	Popliteal Pseudoaneurysm and Arteriovenous Fistula After Arthroscopic Meniscectomy	Case report	No	69	F	Not reported in full text	Meniscectomy	Arthroscopic meniscectomy with femoral/tibial chondroplasty	Not reported in full text	Popliteal artery; two pseudoaneurysms with AVF	Calf pain/swelling; burning/shooting pain; bruit/thrill/pulsatile swelling	Not reported in full text	Angiography; Doppler/CT/MRI discussed	Open repair attempts; Dacron patch failed; further vascular repair; tibial nerve neurolysis	Incomplete symptom resolution; persistent moderate plantar/medial foot pain; cane use	Arteriovenous fistula; tibial nerve symptoms; poor/incomplete outcome	Full-text audit completed against available full text; unresolved fields marked Not reported	High		Meniscal procedure	Popliteal artery	Open surgical vascular treatment	Nerve procedure	Persistent neurologic/functional morbidity or incomplete recovery		Ultrasound not counted as primary diagnostic vascular ultrasound; Angiography confirmatory/planning/treatment-associated or sequence mixed
57	57A	57. Arthroscopy. 1995 Apr. 229-30. Pseudoaneurysm complicating knee arthroscopy.PDF	Aldrich	1995	USA	Arthroscopy	Pseudoaneurysm complicating knee arthroscopy	Case report	No	19	F	Not reported in full text	Meniscectomy	Arthroscopy and partial medial meniscectomy	Prior arthroscopy/partial meniscectomy; superior medial portal involved; additional details limited in scanned PDF/OCR	Superior medial geniculate artery	Persistent knee pain/intermittent effusion; soft mass over previous superomedial portal	10 weeks postop	Arteriogram	Surgical resection	Did well without further problems	Recurrent hemarthrosis/effusion	Full-text audit completed against available full text; unresolved fields marked Not reported	Moderate (scanned PDF)		Meniscal procedure	Superior medial/middle genicular artery	Open surgical vascular treatment	None separately reported	Favorable recovery/resolution without persistent morbidity documented	70	Angiography served as primary/definitive vascular imaging
57	57B	57. Arthroscopy. 1995 Apr. 229-30. Pseudoaneurysm complicating knee arthroscopy.PDF	Aldrich	1995	USA	Arthroscopy	Pseudoaneurysm complicating knee arthroscopy	Case report	No	29	M	Not reported in full text	Meniscectomy	Arthroscopy and partial meniscectomy after twisting injury	Inferolateral portal site mentioned	Recurrent anterior tibial artery	Tender pulsatile mass near inferolateral portal; swelling and blood aspirated	12 days postop	Arteriogram	Surgical ligation	Not reported in full text	Late presentation	Full-text audit completed against available full text; unresolved fields marked Not reported	Moderate (scanned PDF)		Meniscal procedure	Anterior tibial artery	Open surgical vascular treatment	None separately reported	Outcome not reported	12	Angiography served as primary/definitive vascular imaging
58	58	58. Clin Orthop Relat Res. 1993 Oct. 214-7. Pseudoaneurysm after arthroscopy of the knee..PDF	Guy	1993	UK	Clinical Orthopaedics and Related Research	Pseudoaneurysm After Arthroscopy of the Knee	Case report	No	25	M	Not reported in full text	Diagnostic arthroscopy	Diagnostic arthroscopy for ACL rupture/possible medial meniscal tear	Standard anterolateral/anteromedial portals; arthroscopic control; tourniquet/irrigation	Inferior lateral genicular artery	Patellar swelling/anterior knee ache; pulsatile 2-cm anterolateral swelling with bruit	3 weeks postop	Radionuclide angiogram; duplex ultrasound; digital subtraction angiography	Exploration; aneurysm excision; no vessel reconstruction required	Discharged next day; remained well at review	Hemarthrosis absent	Full-text audit completed against available full text; unresolved fields marked Not reported	High (visual extraction from scan)		Other diagnostic/therapeutic arthroscopy	Lateral/inferior genicular artery	Open surgical vascular treatment	None separately reported	Favorable recovery/resolution without persistent morbidity documented	21	Diagnostic vascular ultrasound used; Angiography confirmatory/planning/treatment-associated or sequence mixed
59	59A	59. Clin Orthop Relat Rest. 1993 198-200. Popliteal pseudoaneurysm after arthroscopic meniscetomy..PDF	Ritt	1993	Netherlands	Clinical Orthopaedics and Related Research	Popliteal Pseudoaneurysm After Arthroscopic Meniscectomy	Case report	No	22	M	Not reported in full text	Meniscectomy	Arthroscopic partial lateral meniscectomy for bucket-handle lateral meniscus tear	Tourniquet used; surgery uneventful	Popliteal artery	Increasing pain/swelling in popliteal fossa; pulsatile mass	3 weeks postop	Ultrasound; femoral angiography; DSA post-op	Open resection; autologous short saphenous vein interposition graft	One-year follow-up: normal pulses/BPs; no recurrence		Full-text audit completed against available full text; unresolved fields marked Not reported	High (visual extraction from scan)		Meniscal procedure	Popliteal artery	Open surgical vascular treatment	None separately reported	Favorable recovery/resolution without persistent morbidity documented	21	Diagnostic vascular ultrasound used; Angiography confirmatory/planning/treatment-associated or sequence mixed
59	59B	59. Clin Orthop Relat Rest. 1993 198-200. Popliteal pseudoaneurysm after arthroscopic meniscetomy..PDF	Ritt	1993	Netherlands	Clinical Orthopaedics and Related Research	Popliteal Pseudoaneurysm After Arthroscopic Meniscectomy	Case report	No	20	M	Not reported in full text	Meniscectomy	Diagnostic arthroscopy and partial medial meniscectomy for bucket-handle medial meniscus	Tourniquet control	Popliteal artery	Dull posterior knee pain; pulsatile popliteal mass	Several months postop; exact interval not reported	Ultrasound; later angiography	Resection and bypass grafting using autogenous saphenous vein; distal anastomosis revision	12 months: pain-free, full knee motion, no recurrence, normal pedal pulses		Full-text audit completed against available full text; unresolved fields marked Not reported	High (visual extraction from scan)		Meniscal procedure	Popliteal artery	Open surgical vascular treatment	None separately reported	Favorable recovery/resolution without persistent morbidity documented		Diagnostic vascular ultrasound used; Angiography confirmatory/planning/treatment-associated or sequence mixed
60	60	60. J Trauma. 1986 Jan. 87-9. Popliteal artery pseudoaneurysm following arthroscopy.PDF	Beck	1986	USA	Journal of Trauma	Popliteal Artery Pseudoaneurysm following Arthroscopy	Case report	No	18	M	Baker cyst; prior arthroscopy with cartilage removed	Diagnostic/therapeutic arthroscopy	Arthroscopy of left knee; Baker cyst noted	Not reported	Popliteal artery	Left foot pain/paralysis/pallor; cool mottled foot; absent dorsalis pedis pulse; popliteal mass	Symptoms weeks/months after arthroscopy; presented with acute ischemia	X-ray calcified mass; arteriogram; DSA post-op	Obliteration of aneurysm and reverse saphenous vein bypass graft	Ischemia resolved; Doppler signal returned; graft functioning/patent at follow-up	Foot ischemia/embolization	Full-text audit completed against available full text; unresolved fields marked Not reported	High (visual extraction from scan)		Other diagnostic/therapeutic arthroscopy	Popliteal artery	Open surgical vascular treatment	None separately reported	Favorable recovery/resolution without persistent morbidity documented		Angiography served as primary/definitive vascular imaging
61	61A	61. False aneurysm after arthroscopic meniscectomy. A report of two cases.pdf	Vincent	1990	Canada	Journal of Bone and Joint Surgery	False Aneurysm after Arthroscopic Meniscectomy	Case report	No	30	M	Good general health	Meniscectomy	Arthroscopic medial meniscectomy for displaced bucket-handle medial meniscus tear	Pneumatic tourniquet; 3-mm vertical anteromedial/anterolateral portals; accessory medial portal	Lateral inferior genicular artery	Postoperative sanguineous drainage; painful hemarthrosis	6 days postop hemarthrosis; angiographic diagnosis at clinic visit 1 week later	Angiography	Resection/open surgical management	Rapid symptom resolution after resection (as reported by the article)	Hemarthrosis	Full-text audit completed against available full text; unresolved fields marked Not reported	High		Meniscal procedure	Lateral/inferior genicular artery	Open surgical vascular treatment	None separately reported	Favorable recovery/resolution without persistent morbidity documented	7	Angiography served as primary/definitive vascular imaging
61	61B	61. False aneurysm after arthroscopic meniscectomy. A report of two cases.pdf	Vincent	1990	Canada	Journal of Bone and Joint Surgery	False Aneurysm after Arthroscopic Meniscectomy	Case report	No	23	M	Chronic ACL tear history; no additional comorbidity/coagulopathy reported in full text	Meniscectomy	Diagnostic arthroscopy and partial medial meniscectomy for bucket-handle medial meniscus tear	Pneumatic tourniquet; horizontal anterolateral/anteromedial portals; second medial portal	Descending genicular artery	Postoperative portal swelling; 4 x 4 cm pulsatile mass with thrill/bruit	2 weeks postop	Angiography	Aneurysm resection and feeding vessel ligation	24 months: pain-free, full ROM, no recurrence		Full-text audit completed against available full text; unresolved fields marked Not reported	High		Meniscal procedure	Descending genicular artery/branch	Open surgical vascular treatment	None separately reported	Favorable recovery/resolution without persistent morbidity documented	14	Angiography served as primary/definitive vascular imaging
62	62	62. Pseudoaneurysm of the superior medial genicular artery following knee arthroscopy.pdf	Mufty	2011	Belgium	Knee Surgery, Sports Traumatology, Arthroscopy	Pseudoaneurysm of the superior medial genicular artery following knee arthroscopy	Case report	Original case only; no duplicate cases extracted from reference discussion	45	M	Not reported in full text	Meniscectomy or meniscal procedure	Arthroscopic partial meniscectomy for degenerative medial meniscus damage	Medial portal; partial meniscectomy; other surgical details not reported in full text	Superior medial genicular artery	Postoperative hemarthrosis recurring after two aspirations; pulsating mass near medial portal 3 days after arthroscopy	3 days postop for recognition of pulsating mass; treated one week later	Computed tomography; arterial duplex ultrasound	Surgical exploration with excision of the pseudoaneurysm and ligation of the feeding artery	Two months after arthroscopy, only slight discomfort over the incision	Not reported in full text	Included - added after full text obtained	High	Mufty et al. 2011 added from newly obtained full text. The second uploaded PDF titled as Guermazi 1996 contained Vassallo et al. 1989, which was already included, so no new Guermazi case was added.	Meniscal procedure	Superior medial/middle genicular artery	Open surgical vascular treatment	None separately reported	Favorable recovery/resolution without persistent morbidity documented	3	Diagnostic vascular ultrasound used
63	63	63. Geniculate artery pseudoaneurysm a rare complication of arthroscopic surgery.pdf	Armato	1990	USA	AJR American Journal of Roentgenology	Geniculate Artery Pseudoaneurysm: A Rare Complication of Arthroscopic Surgery	Case report/letter	No	59	M	Prior lateral tibial osteotomy 10 years earlier; degenerative knee disease; no coagulopathy reported	Synovectomy/arthroscopy	Diagnostic arthroscopy; partial lateral synovectomy was terminated after brisk bleeding	Specific portal and tourniquet details not reported	Inferior lateral geniculate artery	Considerable postoperative pain and swelling; bloody knee aspirations at 24 and 48 hours; acute increase in pain and swelling on postoperative day 10	10 days postop	MRI raised vascular concern; selective digital subtraction angiography confirmed the pseudoaneurysm; color Doppler was performed afterward for comparison	No pseudoaneurysm-directed treatment reported; patient was stable at discharge	Stable at discharge; longer-term vascular or functional outcome not reported	Supraventricular tachycardia during aspiration; no active bleeding on angiography	Verified against attached full text	High	Added during peer-review citation audit. Color Doppler was confirmatory after angiography, not the initial diagnostic test.	Synovectomy/debridement/irrigation/cyst procedure	Lateral/inferior genicular artery	No definitive treatment reported	None separately reported	Limited or short-term follow-up; definitive recovery not established	10	Ultrasound not counted as primary diagnostic vascular ultrasound; Angiography confirmatory/planning/treatment-associated or sequence mixed
64	64	64. Pseudoaneurysm of the medial inferior genicular artery following anterior.pdf	Milankov	2006	Serbia and Montenegro	The Knee	Pseudoaneurysm of the medial inferior genicular artery following anterior cruciate ligament reconstruction with hamstring tendon autograft	Case report/short communication	No	37	M	No coagulopathy reported; ACL rupture from soccer injury	ACLR	Arthroscopic ACL reconstruction using quadrupled hamstring tendon autograft	Spinal anesthesia; tourniquet used; knee and operative-wound drains placed	Medial inferior genicular artery	Continuous filling of medial operative-wound drain one hour after surgery; posterior lower leg became swollen, hard, and painful; distal pulses remained present	1 day postop	Doppler examination showed no major-vessel injury; ultrasound showed posterior-compartment hematoma; angiography confirmed medial inferior genicular artery injury and pseudoaneurysm	Revision surgery with hematoma evacuation and ligation of the injured vessel	No postoperative complications; at six months the knee had full range of motion and was stable, and the patient had resumed recreational soccer	None reported after revision surgery	Verified against attached full text	High	Added during peer-review citation audit.	ACL-related procedure	Medial/inferior genicular artery	Open surgical vascular treatment	Hematoma/clot evacuation or drainage	Favorable recovery/resolution without persistent morbidity documented	1	Diagnostic vascular ultrasound used; Angiography confirmatory/planning/treatment-associated or sequence mixed

Methodological Appraisal

The prior binary report-level appraisal table was removed because it treated all publications as case reports and could overstate deficiencies by converting uncertain or non-applicable domains into negative judgments. The revised supplementary appraisal uses design-matched JBI tools, preserves Unclear and Not applicable responses, and is interpreted qualitatively rather than as a score. Item-level JBI critical appraisal results for the 50 single-patient case reports are presented in Table 8. 

**Table 8:** JBI critical appraisal - single-patient case reports (n = 50) JBI appraisal responses were coded as Y (yes), N (no), U (unclear), or NA (not applicable); no numerical quality score was calculated, and appraisal was used qualitatively without determining study eligibility.

Study/report	Q1	Q2	Q3	Q4	Q5	Q6	Q7	Q8
Carroll et al. (2020) [1]	Y	Y	Y	Y	Y	Y	U	Y
Sajjad et al. (2014) [2]	Y	Y	Y	Y	Y	Y	Y	Y
Smoter et al. (2025) [3]	Y	Y	Y	Y	Y	Y	Y	Y
Becher et al. (2008) [4]	Y	Y	Y	Y	Y	Y	Y	Y
Venkatesh et al. (2009) [5]	Y	Y	Y	Y	Y	Y	Y	U
Tozzi et al. (1996) [7]	Y	Y	Y	Y	Y	Y	U	Y
Janssen et al. (2004) [8]	Y	Y	Y	Y	Y	U	Y	Y
Shahrulazua et al. (2014) [9]	Y	Y	Y	Y	Y	Y	Y	Y
Lee et al. (2007) [10]	Y	Y	Y	Y	Y	Y	Y	U
Tsubosaka et al. (2017) [11]	Y	Y	Y	Y	Y	Y	U	Y
Ho et al. (2020) [12]	Y	Y	Y	Y	Y	Y	U	Y
Mello et al. (2011) [13]	Y	Y	Y	Y	Y	Y	Y	Y
Castroviejo et al. (2024) [14]	Y	Y	Y	Y	Y	Y	U	Y
Ambrosia et al. (2015) [15]	Y	Y	Y	U	U	U	Y	Y
Evans et al. (2000) [16]	Y	Y	Y	Y	Y	Y	Y	Y
Xu et al. (2017) [17]	Y	Y	Y	Y	U	U	Y	Y
Lee et al. (2014) [18]	U	Y	Y	Y	U	U	Y	Y
Tam et al. (2014) [19]	Y	Y	Y	Y	Y	U	Y	Y
Joshi et al. (2019) [21]	Y	Y	Y	U	Y	Y	Y	Y
Akan et al. (2025) [22]	Y	Y	Y	Y	Y	Y	Y	Y
Joshi et al. (2014) [23]	Y	Y	Y	Y	Y	Y	Y	U
Jin et al. (2015) [24]	Y	Y	Y	Y	Y	Y	Y	Y
Rachakonda et al. (2015) [25]	Y	Y	Y	Y	Y	Y	U	Y
Puig et al. (2007) [27]	Y	U	U	Y	Y	Y	Y	Y
Alserr et al. (2014) [28]	Y	Y	Y	Y	Y	Y	Y	Y
AbuRahma et al. (2013) [29]	Y	Y	Y	Y	Y	Y	U	Y
Vassallo et al. (1989) [30]	Y	Y	Y	Y	Y	Y	Y	Y
Hussein et al. (1998) [31]	Y	U	U	Y	Y	Y	U	Y
Manning et al. (1987) [32]	Y	Y	Y	Y	Y	Y	Y	Y
Mullen et al. (2001) [33]	Y	U	Y	Y	Y	Y	Y	Y
Guy et al. (1993) [34]	Y	Y	Y	Y	Y	Y	Y	U
Beck et al. (1986) [36]	Y	Y	Y	Y	Y	Y	Y	U
Bhamidipati et al. (2024) [38]	Y	Y	Y	Y	Y	Y	U	Y
Glanz et al. (2020) [39]	U	Y	Y	Y	Y	Y	U	Y
Barrio Piqueras et al. (2025) [40]	Y	Y	Y	Y	Y	Y	Y	Y
Lee et al. (2009) [41]	Y	Y	Y	Y	Y	Y	Y	Y
Gliatis et al. (2023) [42]	Y	Y	Y	Y	Y	Y	Y	Y
Ben Romdhane et al. (1991) [46]	Y	Y	Y	Y	Y	U	Y	Y
Kiss et al. (2001) [47]	Y	Y	Y	Y	Y	Y	Y	Y
Mufty et al. (2011) [48]	Y	Y	Y	Y	Y	Y	Y	Y
Oversier et al. (2018) [49]	Y	Y	Y	Y	Y	Y	U	Y
Iqbal et al. (2025) [50]	Y	Y	Y	Y	Y	Y	U	Y
Stieven Filho et al. (2015) [51]	Y	Y	Y	Y	Y	Y	U	Y
Audenaert et al. (2003) [52]	Y	Y	Y	Y	Y	Y	Y	Y
Leung et al. (2020) [53]	Y	Y	Y	Y	Y	Y	Y	Y
Simmons et al. (2022) [54]	Y	Y	Y	Y	Y	Y	Y	Y
Ellis (2018) [55]	Y	Y	Y	Y	Y	Y	Y	U
Sarrosa et al. (1997) [56]	Y	U	Y	Y	Y	Y	U	Y
Armato & Czarnecki (1990) [58]	Y	Y	Y	Y	NA	Y	Y	Y
Milankov et al. (2006) [59]	Y	Y	Y	Y	Y	Y	Y	Y

Item-level JBI critical appraisal results for the six multi-patient reports are presented in Table 9.

**Table 9:** JBI critical appraisal - multi-patient reports/case series (n = 6) JBI appraisal responses were coded as Y (yes), N (no), U (unclear), or NA (not applicable); no numerical quality score was calculated, and appraisal was used qualitatively without determining study eligibility.

Study/report	Q1	Q2	Q3	Q4	Q5	Q6	Q7	Q8	Q9	Q10
Carlin et al. (2001) [6]	U	Y	Y	U	U	Y	Y	Y	U	NA
Bonfim et al. (2023) [20]	U	Y	Y	U	U	Y	Y	Y	U	NA
Hilborn et al. (1994) [26]	U	Y	Y	U	U	Y	Y	U	U	NA
Ritt et al. (1993) [35]	U	Y	Y	U	U	Y	Y	Y	U	NA
Vincent et al. (1990) [37]	U	Y	Y	U	U	Y	Y	Y	U	NA
Aldrich et al. (1995) [57]	U	Y	Y	U	U	Y	Y	U	U	NA

Procedure and Injured Vessel Distribution

Using the revised mutually exclusive primary-procedure classification, meniscal procedures accounted for 25/62 cases (40.3%), isolated ACL-related procedures for 19/62 (30.6%), other diagnostic/therapeutic arthroscopy for 8/62 (12.9%), synovectomy/debridement/irrigation/cyst procedures for 6/62 (9.7%), PCL/combined cruciate procedures for 3/62 (4.8%), and combined ACL-meniscal procedures for 1/62 (1.6%). The distribution of mutually exclusive primary arthroscopic procedure categories among the 62 cases is illustrated in Figure 2.

**Figure 2:**
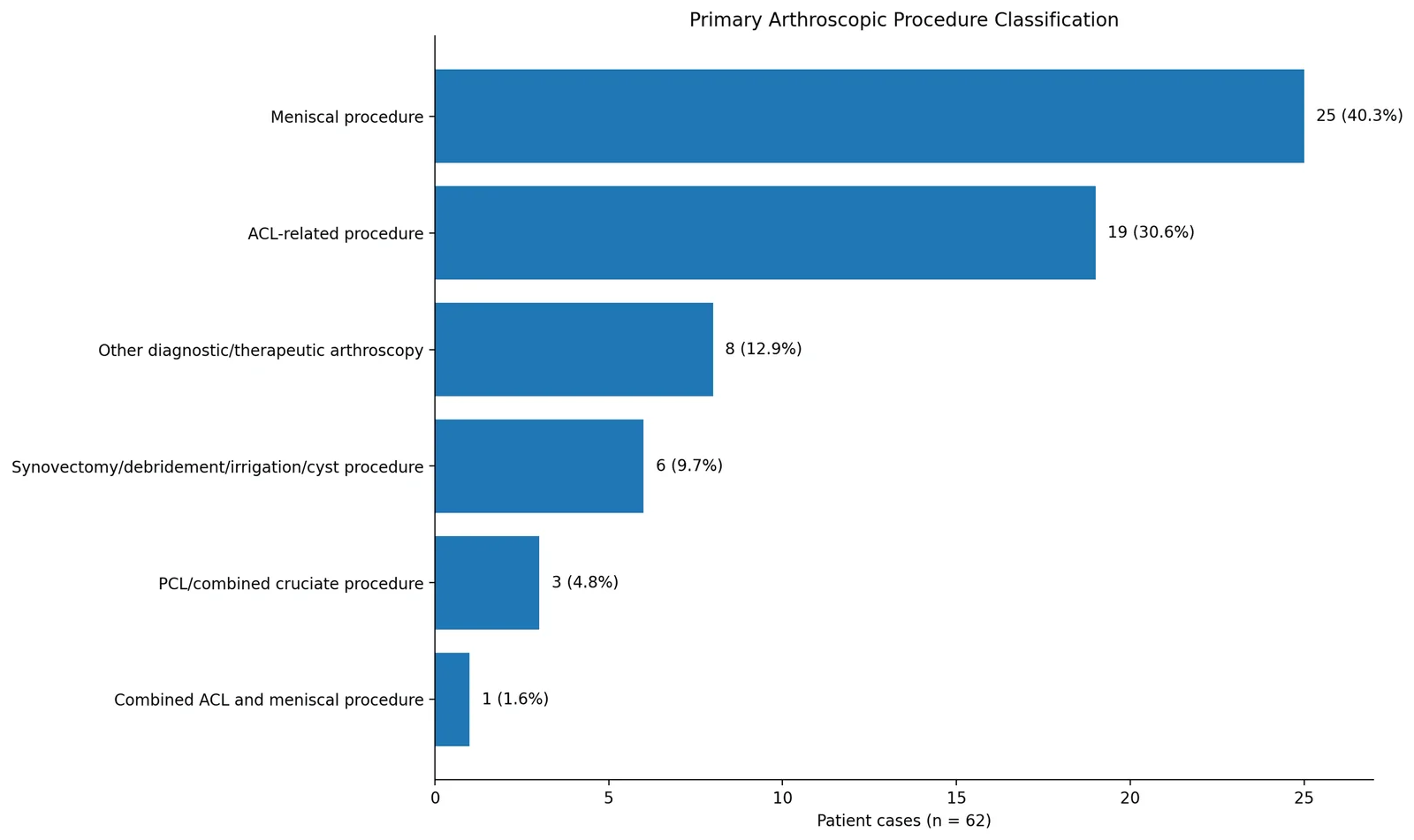
Mutually exclusive primary arthroscopic procedure classification among 62 patient cases ACL: Anterior cruciate ligament; PCL: posterior cruciate ligament

After source terminology was verified and grouped consistently, the popliteal artery was the most frequent injured-vessel group (26/62, 41.9%). Medial/inferior genicular branches accounted for 9/62 (14.5%) and lateral/inferior genicular branches for 6/62 (9.7%). The remaining standardized groups are shown in Figure 3.

**Figure 3:**
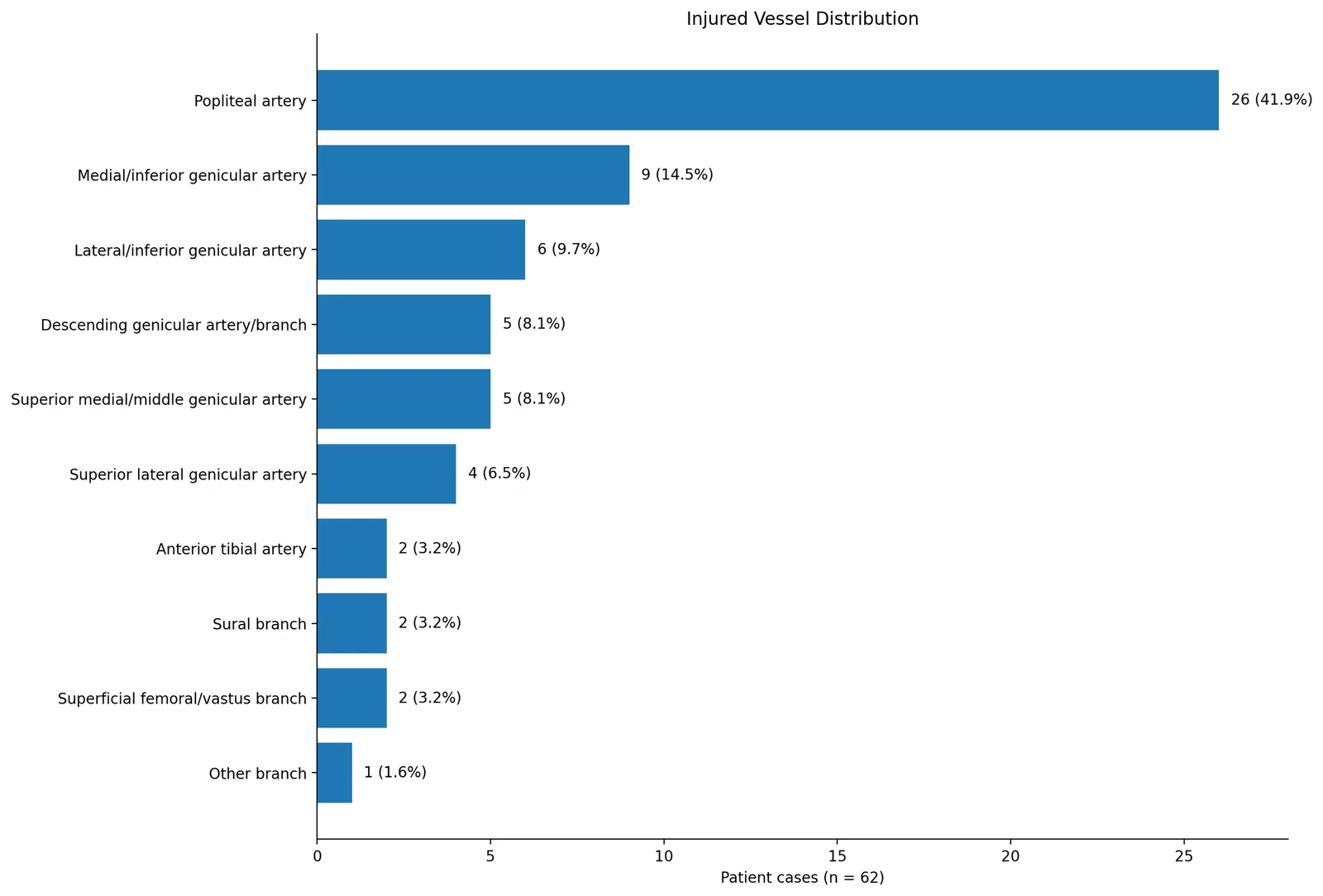
Standardized injured-vessel distribution. The source-reported “superior middle genicular artery” terminology is retained in the raw dataset and grouped with superior medial/middle genicular terminology for synthesis

Clinical Presentation and Timing

Presentation categories were descriptive and could overlap within a patient. Swelling, effusion, edema, or hemarthrosis was documented in 42/62 cases (67.7%), pain in 35/62 (56.5%), and a pulsatile mass, bruit, or thrill in 33/62 (53.2%). Bleeding/hematoma/ecchymosis or recurrent hemarthrosis was documented in 15/62 (24.2%), neurologic findings in 10/62 (16.1%), a DVT-like presentation in 4/62 (6.5%), and compartment-syndrome/ischemic features in 7/62 (11.3%).

An explicitly quantifiable interval from the index arthroscopic procedure to vascular diagnosis or first definite pseudoaneurysm recognition was available in 49/62 cases. The median interval was 14 days, with a range of 1 to 365 days. Thirteen cases had missing or non-quantifiable timing and were not converted from vague wording.

Imaging and Diagnosis

The included reports frequently used more than one imaging modality, so imaging categories are not mutually exclusive. Noninvasive vascular ultrasound was counted as diagnostic only when it was used to evaluate the vascular lesion; ultrasound used solely for aspiration guidance, treatment guidance, or follow-up was not counted as diagnostic vascular ultrasound. Catheter angiography was often performed after ultrasound, CT angiography, or MR angiography, or in conjunction with embolization. It is therefore described in the patient-level dataset by clinical role rather than interpreted as a uniform initial diagnostic test. The newly added Milankov case illustrates this sequence: Doppler and ultrasound preceded angiographic confirmation [59]. In the Armato case, color Doppler was performed after digital subtraction angiography for comparison and was not classified as the initial diagnostic test [58].

Treatment and Outcomes

Primary definitive treatment was assigned once per case. Open surgical vascular treatment was the primary approach in 30/62 cases (48.4%); endovascular embolization/coiling in 14/62 (22.6%); ultrasound-guided thrombin injection in 6/62 (9.7%); covered stent or other endovascular repair in 5/62 (8.1%); conservative observation in 2/62 (3.2%); vascular treatment with approach not reported in 4/62 (6.5%); and no definitive pseudoaneurysm-directed treatment was reported in 1/62 (1.6%). Adjunctive procedures were not mixed into these categories. Three patients underwent fasciotomy/decompression, and hematoma drainage/evacuation or nerve procedures were separately recorded when reported.

Outcome categories were mutually exclusive. Favorable recovery or vascular resolution without persistent morbidity was documented in 42/62 cases (67.7%). Persistent neurologic/functional morbidity or incomplete recovery was documented in 4/62 (6.5%). Ten cases (16.1%) had only limited or short-term follow-up that did not establish definitive recovery, and 6/62 (9.7%) did not report an outcome. These categories replace the prior composite “favorable outcome” measure. Table 10 provides a descriptive summary of patient characteristics, diagnostic timing, presenting features, treatments, and outcomes. 

**Table 10:** Descriptive summary. *Presentation categories can overlap. Primary procedure, primary treatment, and outcome categories are mutually exclusive. No confidence intervals are reported.

Domain	Variable	Frequency	Descriptive summary
Demographics	Age reported	60/62	Exact numeric age in 59/62; median 31 years; range 8-84
Demographics	Sex reported	60/62	46 male; 14 female
Timing	Quantifiable time to diagnosis/recognition	49/62	Median 14 days; range 1-365; 13 missing/non-quantifiable
Primary procedure	Meniscal procedure	25/62	40.3%
Primary procedure	ACL-related procedure	19/62	30.6%
Vessel	Popliteal artery	26/62	41.9%
Vessel	Medial/inferior genicular artery	9/62	14.5%
Presentation*	Swelling/effusion/edema/hemarthrosis	42/62	67.7%
Presentation*	Pain	35/62	56.5%
Presentation*	Pulsatile mass/bruit/thrill	33/62	53.2%
Primary treatment	Open surgical vascular treatment	30/62	48.4%
Primary treatment	Endovascular embolization/coiling	14/62	22.6%
Primary treatment	Ultrasound-guided thrombin injection	6/62	9.7%
Outcome	Favorable recovery/resolution without persistent morbidity	42/62	67.7%
Outcome	Persistent neurologic/functional morbidity or incomplete recovery	4/62	6.5%
Outcome	Limited/short-term follow-up	10/62	16.1%
Outcome	Not reported	6/62	9.7%

Counts and percentages for each primary procedure category, injured-vessel group, and final outcome category are presented in Table 11.

**Table 11:** Quantitative summary Time to diagnosis was quantifiable in 49 cases, with a median of 14 days (range, 1–365 days); timing was missing or unquantifiable in 13 cases.

Domain	Category	Count	Percent of 62 cases
Primary procedure	Meniscal procedure	25	40.3%
Primary procedure	ACL-related procedure	19	30.6%
Primary procedure	Other diagnostic/therapeutic arthroscopy	8	12.9%
Primary procedure	Synovectomy/debridement/irrigation/cyst procedure	6	9.7%
Primary procedure	PCL/combined cruciate procedure	3	4.8%
Primary procedure	Combined ACL and meniscal procedure	1	1.6%
Vessel	Popliteal artery	26	41.9%
Vessel	Medial/inferior genicular artery	9	14.5%
Vessel	Lateral/inferior genicular artery	6	9.7%
Vessel	Descending genicular artery/branch	5	8.1%
Vessel	Superior medial/middle genicular artery	5	8.1%
Vessel	Superior lateral genicular artery	4	6.5%
Vessel	Anterior tibial artery	2	3.2%
Vessel	Sural branch	2	3.2%
Vessel	Superficial femoral/vastus branch	2	3.2%
Vessel	Other branch	1	1.6%
Outcome	Favorable recovery/resolution without persistent morbidity documented	42	67.7%
Outcome	Persistent neurologic/functional morbidity or incomplete recovery	4	6.5%
Outcome	Limited or short-term follow-up; definitive recovery not established	10	16.1%
Outcome	Outcome not reported	6	9.7%

Discussion

The main clinical message is not that one procedure or one vessel defines this complication, but that vascular injury can emerge from several arthroscopic mechanisms and can initially resemble common postoperative problems. A mutually exclusive procedure classification clarifies that meniscal and ACL-related procedures account for the largest shares of the published cases without double-counting combined procedures. The vessel analysis likewise shows a substantial burden of genicular and other branch-vessel injuries in addition to popliteal lesions.

Diagnostic delay remains a practical concern. The median quantifiable interval was two weeks, but the range extended to one year. The case literature shows why: distal pulses can remain present, nonvascular diagnoses are frequently considered first, and early ultrasound may be negative or may be performed for another purpose. These observations support careful reassessment when postoperative swelling, hemarthrosis, focal pain, a new pulsatile finding, neurologic deficit, or ischemic features recur or progress rather than resolving as expected.

The revised imaging analysis also avoids treating angiography and ultrasound as single-role variables. Duplex ultrasound may be the first vascular test in an accessible lesion, CT angiography may map deeper anatomy or clarify a competing diagnosis, and catheter angiography can serve as confirmation, treatment planning, or treatment itself. A report that mentions angiography therefore does not imply that angiography was the first diagnostic study, and ultrasound used only for aspiration or follow-up does not represent diagnostic vascular ultrasound.

Treatment should similarly be interpreted as anatomy- and severity-dependent rather than as a historical contest between open and endovascular techniques. The included reports describe both open vascular reconstruction/ligation and less invasive embolization, thrombin injection, or stent-based treatment. The available evidence does not support formal comparisons among these approaches because case selection, vessel caliber, rupture, arteriovenous fistula, neurologic compression, and local expertise differ substantially across reports. The prior manuscript wording suggesting a temporal treatment shift has therefore been softened rather than converted into an unsupported era comparison.

The two newly included reports reinforce the need for branch-vessel awareness. Armato and Czarnecki described an inferior lateral geniculate pseudoaneurysm after partial lateral synovectomy, with MRI and digital subtraction angiography establishing the diagnosis [58]. Milankov et al. described a medial inferior genicular pseudoaneurysm after hamstring-autograft ACL reconstruction; distal pulses remained present, noninvasive imaging preceded angiographic confirmation, and ligation led to full functional recovery at six months [59]. The third report identified during review could not be retrieved, so it was not used to infer patients, vessels, treatments, or outcomes. Table 12 presents a pragmatic, literature-informed framework for recognizing, evaluating, and managing suspected pseudoaneurysm after knee arthroscopy; this framework has not been validated.

**Table 12:** Pragmatic, literature-informed framework derived from case reports. This is not a validated diagnostic or treatment algorithm.

Pragmatic step	Literature-informed clinical application
Recognize a concerning postoperative pattern	Recurrent/progressive pain or swelling, repeated hemarthrosis, focal pulsatile mass/bruit/thrill, new neurologic deficit, ischemic change, or symptoms out of proportion to the expected course.
Perform focused vascular and neurologic examination	Compare pulses, capillary refill, temperature, swelling, focal mass, bruit/thrill, motor function, and sensation. Normal distal pulses do not fully exclude branch-vessel injury.
Consider vascular imaging	Duplex ultrasound is useful for accessible lesions; CTA can define deeper anatomy and competing diagnoses. Choice depends on clinical context and local expertise.
Escalate when suspicion persists	Catheter angiography can confirm anatomy and may permit embolization when appropriate. Persistent symptoms despite nondiagnostic initial testing warrant reassessment.
Match treatment to anatomy/severity	Open repair/ligation/reconstruction, embolization, thrombin injection, stent-based treatment, or observation may be appropriate in selected cases; evidence is case-based and not comparative.

Limitations

This review is limited by the underlying evidence base. Case reports and small case series are highly susceptible to publication bias and selective reporting; severe, unusual, technically interesting, or successfully treated cases are more likely to be published, while mild, spontaneously thrombosing, conservatively managed, or unrecognized cases are likely underrepresented. Therefore, the observed frequencies cannot estimate incidence, risk, or comparative treatment effectiveness.

Reporting quality and follow-up were inconsistent, and some older reports did not provide sufficient detail to classify timing, imaging sequence, or final functional outcome. The literature also spans different arthroscopic techniques and eras without a common denominator. A formal protocol was not prospectively registered. The initial reference-list search was not count-logged separately, so a retrospective hand-search count was not invented; the peer-review citation audit is transparently reported as an add-on pathway. One peer-review-identified publication could not be retrieved and was not included.

Although no full-text report was excluded solely for language, requiring sufficient patient-level information for reliable extraction can still introduce language-related selection bias. No translation was required among the full texts ultimately assessed, but non-English or difficult-to-access reports may remain underrepresented. Finally, the design-matched JBI appraisal is descriptive and does not overcome the intrinsic selection and reporting limitations of case-based literature.

## Conclusions

Iatrogenic pseudoaneurysm after arthroscopic knee procedures is rare but clinically important because it can resemble routine postoperative complications and may be diagnosed days to months after surgery. Published cases involve both the popliteal artery and multiple peri-genicular branches. Recurrent or progressive swelling, hemarthrosis, focal pulsatility, neurologic change, ischemic features, or symptoms out of proportion to the expected course should prompt consideration of vascular imaging. Treatment must be individualized to the injured vessel, lesion characteristics, associated complications, and local expertise. Because the evidence is exclusively case-based, these findings should be used as a literature-informed warning framework rather than as estimates of incidence or as a validated diagnostic or treatment algorithm.
